# Correction: Comparative analysis of various clinical specimens in detection of SARS-CoV-2 using rRT-PCR in new and follow up cases of COVID-19 infection: Quest for the best choice

**DOI:** 10.1371/journal.pone.0253355

**Published:** 2021-06-10

**Authors:** Kuldeep Sharma, Pragya Agarwala, Deepa Gandhi, Anuniti Mathias, Priyanka Singh, Somya Sharma, Sanjay Singh Negi, Anudita Bhargava, Padma Das, Ujjwala Gaikwad, Archana Wankhede, Ajoy Behra, Nitin M. Nagarkar

The second author’s name is spelled incorrectly. The correct name is: Pragya Agarwala. The correct citation is: Sharma K, Agarwala P, Gandhi D, Mathias A, Singh P, et al. (2021) Comparative analysis of various clinical specimens in detection of SARS-CoV-2 using rRT-PCR in new and follow up cases of COVID-19 infection: Quest for the best choice. PLOS ONE 16(4): e0249408. https://doi.org/10.1371/journal.pone.0249408
