# Supplementary material for: Comparative analysis of various clinical specimens in detection of SARS-CoV-2 using rRT-PCR in new and follow up cases of COVID-19 infection: Quest for the best choice
Source: PLoS One. 2021 Apr 5;16(4):e0249408. doi: 10.1371/journal.pone.0249408 (PMC8021168; doi:10.1371/journal.pone.0249408)
Supplement: Attachment — Submitted filename: PONE-D-20-38439_R2.pdf [file pone.0249408.s004.pdf]

PLOS ONE

Comparative analysis of various clinical specimens in detection of SARS-CoV-2 using rRT-PCR in new and follow up cases of COVID-19 infection: quest for the best choice

--Manuscript Draft--

Manuscript Number:	PONE-D-20-38439R2
Article Type:	Research Article
Full Title:	Comparative analysis of various clinical specimens in detection of SARS-CoV-2 using rRT-PCR in new and follow up cases of COVID-19 infection: quest for the best choice
Short Title:	Evaluation of multivaried clinical specimens in diagnosis of COVID-19
Corresponding Author:	Sanjay Singh Negi, Ph.D.<br>All India Institute of Medical Sciences - Raipur<br>raipur, Chhattisgarh INDIA
Keywords:	SARS-CoV-2, nCoV, rRT-PCR, combined swab
Abstract:	An appropriate specimen is of paramount importance in Real Time reverse transcription-polymerase chain reaction (rRT-PCR) based diagnosis of novel coronavirus (nCoV) disease (COVID-19). Thus, it's pertinent to evaluate various diversified clinical specimens' diagnostic utility in both diagnosis and follow-up of COVID-19. A total of 924 initial specimens from 130 COVID-19 symptomatic cases before initiation of treatment and 665 follow up specimens from 15 randomly selected cases comprising of equal number of nasopharyngeal swab (NPS), oropharyngeal swab (OPS), combined NPS and OPS (Combined swab), sputum, plasma, serum and urine were evaluated by rRT-PCR. Combined swabs showed a positivity rate of 100 % followed by NPS (91.5%), OPS (72.3%), sputum (63%), while nCoV was found undetected in urine, plasma and serum specimens. The lowest cycle threshold (Ct) values of targeted genes E, ORF1b and RdRP of 10.56, 10.14 and 12.26 and their lowest average Ct values were found in combined swab which indicates high viral load in combined swab among all other specimen types. Analysis of 665 follow-up multi-varied specimens also showed combined swab as the last specimen among all specimen types to become negative, after an average 6.6 (range 4-10) days post-treatment, having lowest (15.48) and average(29.96) Ct values of ORF1b respectively indicating posterior nasopharyngeal tract as primary nCoV afflicted site with high viral load. The combined swab thus, may be recommendation as a more appropriate specimen for both diagnosis and monitoring of COVID-19 treatment by rRT-PCR for assessing virus clearance to help physicians in taking evidence-based decision before discharging patients. Implementing combined swabs globally will definitely help in management and control of the pandemic, as it is the need of the hour.
Order of Authors:	Sanjay Singh Negi, Ph.D.
	Kuldeep Sharma
	Pragya Aggarwala
	Deepa Gandhi
	Anuniti Mathias
	Priyanka Singh
	Somya Sharma
	Anudita Bhargava
	Padma Das
	Ujjwala Gaikwad
	Archana Wankhede
	Ajoy Behra
	Nitin M Nagarkar

Powered by Editorial Manager® and ProduXion Manager® from Aries Systems Corporation

**Opposed Reviewers:**	Arvind Rai, MD<br>Joint Director, National Centre for Disease Control, Delhi<br>arvindrai62@yahoo.in<br>Vast experience of working in Virology. Eminent Virologist in India.
	Syed Tazeen Pasha, Ph.D<br>Joint Director, National Centre for Disease Control, Delhi<br>negidr@yahoo.co.in<br>Eminent Virologist in India
**Response to Reviewers:**	We have tried to adequately address all the suggestions and comments made by reviewer and journal editorial office.
**Additional Information:**	
**Question**	**Response**
**Financial Disclosure**<br><br>Enter a financial disclosure statement that describes the sources of funding for the work included in this submission. Review the submission guidelines for detailed requirements. View published research articles from *PLOS ONE* for specific examples.<br><br>This statement is required for submission and **will appear in the published article** if the submission is accepted. Please make sure it is accurate.<br><br>**Unfunded studies**<br>Enter: *The author(s) received no specific funding for this work.*<br><br>**Funded studies**<br>Enter a statement with the following details:<br>• Initials of the authors who received each award<br>• Grant numbers awarded to each author<br>• The full name of each funder<br>• URL of each funder website<br>• Did the sponsors or funders play any role in the study design, data collection and analysis, decision to publish, or preparation of the manuscript?<br>• **NO** - Include this sentence at the end of your statement: *The funders had no role in study design, data collection and analysis, decision to publish, or preparation of the manuscript.*<br>• **YES** - Specify the role(s) played.<br><br>* typeset	This study was supported by AIIMS, Raipur, Chhattisgarh (AIIMS/Raipur/Admin/110).
**Competing Interests**	The authors have declared that no competing interests exist.

Use the instructions below to enter a competing interest statement for this submission. On behalf of all authors, disclose any competing interests that could be perceived to bias this work—acknowledging all financial support and any other relevant financial or non-financial competing interests.<br><br>This statement **will appear in the published article** if the submission is accepted. Please make sure it is accurate. View published research articles from *PLOS ONE* for specific examples.<br><br>**NO authors have competing interests**<br><br>Enter: *The authors have declared that no competing interests exist.*<br><br>**Authors with competing interests**<br><br>Enter competing interest details beginning with this statement:<br><br>*I have read the journal's policy and the authors of this manuscript have the following competing interests: [insert competing interests here]*<br><br>* typeset	
**Ethics Statement**<br><br>Enter an ethics statement for this submission. This statement is required if the study involved:<br><br>• Human participants<br>• Human specimens or tissue<br>• Vertebrate animals or cephalopods<br>• Vertebrate embryos or tissues<br>• Field research<br><br>Write "N/A" if the submission does not require an ethics statement.<br><br>General guidance is provided below. Consult the submission guidelines for detailed instructions. **Make sure that all**	Study is approved form Institutional Ethical committee.<br>Name of committe: IEC-AIIMS, Riapur.<br>Approval number: AIIMSRPR/IEC/2020/536

Powered by Editorial Manager® and ProduXion Manager® from Aries Systems Corporation

**information entered here is included in the Methods section of the manuscript.**<br><br>**Format for specific study types**<br><br>**Human Subject Research (involving human participants and/or tissue)**<br>• Give the name of the institutional review board or ethics committee that approved the study<br>• Include the approval number and/or a statement indicating approval of this research<br>• Indicate the form of consent obtained (written/oral) or the reason that consent was not obtained (e.g. the data were analyzed anonymously)<br><br>**Animal Research (involving vertebrate animals, embryos or tissues)**<br>• Provide the name of the Institutional Animal Care and Use Committee (IACUC) or other relevant ethics board that reviewed the study protocol, and indicate whether they approved this research or granted a formal waiver of ethical approval<br>• Include an approval number if one was obtained<br>• If the study involved *non-human primates*, add *additional details* about animal welfare and steps taken to ameliorate suffering<br>• If anesthesia, euthanasia, or any kind of animal sacrifice is part of the study, include briefly which substances and/or methods were applied<br><br>**Field Research**<br><br>Include the following details if this study involves the collection of plant, animal, or other materials from a natural setting:<br>• Field permit number<br>• Name of the institution or relevant body that granted permission	
**Data Availability**<br><br>Authors are required to make all data underlying the findings described fully available, without restriction, and from the time of publication. PLOS allows rare exceptions to address legal and ethical	No - some restrictions will apply

concerns. See the PLOS Data Policy and FAQ for detailed information.<br><br>A Data Availability Statement describing where the data can be found is required at submission. Your answers to this question constitute the Data Availability Statement and **will be published in the article**, if accepted.<br><br>**Important:** Stating 'data available on request from the author' is not sufficient. If your data are only available upon request, select 'No' for the first question and explain your exceptional situation in the text box.<br><br>Do the authors confirm that all data underlying the findings described in their manuscript are fully available without restriction?	
**Describe where the data may be found in full sentences. If you are copying our sample text, replace any instances of XXX with the appropriate details.**<br><br>• If the data are **held or will be held in a public repository**, include URLs, accession numbers or DOIs. If this information will only be available after acceptance, indicate this by ticking the box below. For example: *All XXX files are available from the XXX database (accession number(s) XXX, XXX.*).<br>• If the data are all contained **within the manuscript and/or Supporting Information files**, enter the following: *All relevant data are within the manuscript and its Supporting Information files.*<br>• If neither of these applies but you are able to provide **details of access elsewhere**, with or without limitations, please do so. For example:<br><br>*Data cannot be shared publicly because of [XXX]. Data are available from the XXX Institutional Data Access / Ethics Committee (contact via XXX) for researchers who meet the criteria for access to confidential data.*	The study contains the potential information of identity of the patients and thus cannot be shared on direct request to corresponding author. Any request for the data other than shown in the paper requires an official institutional request to IEC, AIIMS, Raipur to the address mentioned below:<br><br>The Secretary Institutional Ethical Committee(IEC),<br>Room No. 2103, 2nd floor,<br>Medical College Complex, Gate No. 5,<br>All India Institute of Medical Sciences(AIIMS), Raipur<br>Chhattisgarh, India-492099.<br>Phone NO.: +91 771-2577231<br>Fax No. : +91 771-2572999<br>Mail id: iec@aiimsraipur.edu.in

The data underlying the results presented in the study are available from (include the name of the third party and contact information or URL).<br>• This text is appropriate if the data are owned by a third party and authors do not have permission to share the data.<br><br>* typeset	
Additional data availability information:	

Dated:08.02.2021

To,

Editor

PLOS One

Subject: Ethical restriction on sharing of data.

Dear Sir,

The present study entitles "***Comparative analysis of various clinical specimens in detectionof SARS-CoV-2 using rRT-PCR in new and follow up cases of COVID-19 infection: quest for the best choice***" has been approved by the Institutional Ethical Committee(IEC), All India Institute of Medical Sciences(AIIMS), Raipur, Chhattisgarh. The study contains the potential information of identity of the patients and thus cannot be shared on direct request to corresponding author. Any request for the data other than shown in the manuscript requires an official institutional request to IEC, AIIMS, Raipur on the address mentioned as under.

The Secretary
Institutional Ethical Committee(IEC),
Room No. 2103, 2nd floor,
Medical College Complex, Gate No. 5,
All India Institute of Medical Sciences(AIIMS), Raipur
Chhattisgarh, India-492099.
Phone NO.: +91 771-2577231
Fax No. : +91 771-2572999
Mail id: iec@aiimsraipur.edu.in

Thus, it is requested to update our Data Availability statement on our behalf to reflect the specific information that data cannot be shared on direct request to corresponding author.

Thanking you

Sincerely yours

Dr Sanjay Singh Negi

Associate Professor

Department of Microbiology

AIIMS,Raipur

Dated:08.03.2021

To,

Editor

PLOS One

Subject: Re-revised manuscript as per journal requirement.

Dear Sir,

This is kindly submitted that the present study entitles "***Comparative analysis of various clinical specimens in detectionof SARS-CoV-2 using rRT-PCR in new and follow up cases of COVID-19 infection: quest for the best choice***" has been re-revised as per the journal suggestion uploaded in the portal. All the changes were highlighted in red.

Thanking you

Sincerely yours

Dr Sanjay Singh Negi

Associate Professor

Department of Microbiology

AIIMS,Raipur

Revised Manuscript

Click here to access/download;Manuscript;Revised manuscript.doc

Comparative analysis of various clinical specimens in detection of SARS-CoV-2 using rRT-PCR in new and follow up cases of COVID-19 infection: quest for the best choice

Authors: Kuldeep Sharma[a,1], Pragya Aggarwala[a,1], Deepa Gandhi[a,1], Anuniti Mathias[a,1], Priyanka Singh[1], Saumya Sharma[1], Sanjay Singh Negi[1#], Anudita Bhargava[1], Padma Das[1], Ujjwala Gaikwad[1], Wankhede A[1], Behra A[2], Nitin M Nagarkar[3].

# Corresponding author

Dr. Sanjay Singh Negi
Associate Professor
Department of Microbiology
AIIMS, Raipur

[a] Kuldeep Sharma, Pragya Aggarwala, Deepa Gandhi, Anuniti Mathias contributed equally to this work as co-first authors.

[1] Microbiology Department, AIIMS, Raipur, Chhattisgarh.

[2] COVID Nodal Officer & Associate Professor, Department of Pulmonary Medicine, AIIMS, Raipur, Chhattisgarh.

[3] Director, AIIMS, Raipur, Chhattisgarh.

Running title: Evaluation of multivaried clinical specimens in diagnosis of COVID-19.

Abstract

An appropriate specimen is of paramount importance in Real Time reverse transcription-polymerase chain reaction (rRT-PCR) based diagnosis of novel coronavirus (nCoV) disease (COVID-19). Thus, it's pertinent to evaluate various diversified clinical specimens' diagnostic utility in both diagnosis and follow-up of COVID-19. A total of 924 initial specimens from 130 COVID-19 symptomatic cases before initiation of treatment and 665 follow up specimens from 15 randomly selected cases comprising of equal number of nasopharyngeal swab (NPS), oropharyngeal swab (OPS), combined NPS and OPS (Combined swab), sputum, plasma, serum and urine were evaluated by rRT-PCR. Combined swabs showed a positivity rate of 100 % followed by NPS (91.5%), OPS (72.3%), sputum (63%), while nCoV was found undetected in urine, plasma and serum specimens. The lowest cycle threshold (Ct) values of targeted genes *E*, *ORF1b* and *RdRP* ~~of~~ 10.56, 10.14 and 12.26 and their lowest average Ct values were found in combined swab which indicates high viral load in combined swab among all other specimen types. Analysis of 665 follow-up multi-varied specimens also showed combined swab as the last specimen among all specimen types to become negative, after an average 6.6 (range 4-10) days post-treatment, having lowest (15.48) and average(29.96) Ct values of *ORF1b* respectively indicating posterior nasopharyngeal tract as primary nCoV afflicted site with high viral load. The combined swab thus, may be recommendation as a more appropriate specimen for both diagnosis and monitoring of COVID-19 treatment by rRT-PCR for assessing virus clearance to help physicians in taking evidence-based decision before discharging patients. Implementing combined swabs globally will definitely help in management and control of the pandemic, as it is the need of the hour.

Key words: SARS-CoV-2, nCoV, rRT-PCR, combined swab.

1. Introduction

SARS-CoV-2, a name given to a novel coronavirus (nCoV) by the International Committee of Taxonomy of Viruses (ICTV), was first reported in December 2019 from Wuhan, China. Since then, it has posed a devastating looming threat to the world, as around 216 countries and territories are so far affected by the virus causing the infection named COVID-19 [1]. Till the date of reporting on 11.11.2020, 50,810,763 were infected, and 1,263,844 succumbed worldwide to the infection [2]. India is the second most affected country after USA, with 8,636,011 confirmed cases and 127,571 deaths as of 11.11.2020 [2]. The disease can occur in any age-group, being more complicated and life-threatening in patients of the older age group and those with underlying co-morbid conditions such as diabetes, hypertension, cardiovascular and cerebrovascular disease [3-5]. The early clinical presentation of the COVID-19 varies from entirely asymptomatic to severely symptomatic. In India, more than 70% of the laboratory-confirmed cases are asymptomatic [3]. In symptomatic patients, the clinical manifestation includes fever, myalgia, dry cough, fatigue, productive cough, shortness of breath, chest pain, loss of taste and smell, etc. The radiological finding of ground-glass opacities on chest X-ray is one of the prominent observations [4,5]. Since SARS-CoV-2 has a high human-to-human transmissibility rate, early diagnosis, immediate isolation and early treatment of positive patients are key to successful management of the pandemic by preventing its spread to others. Since testing is the corner stone of managing the COVID-19 pandemic, highly sensitive and specific testing is essentially required for early identification of not only the symptomatic cases but also of the asymptomatic cases and their close high-risk contacts, which would potentially break the chain of transmission of COVID-19 infection, which otherwise appears unstoppable at the moment.

Among various viral diagnostic modalities, virus isolation does not appear practically feasible for COVID-19 since it requires Biosafety Level (BSL)-3 laboratory, high technical expertise

and longer turn-around time of 3-5 days to identify cytopathic effect in specific cell lines such as Vero E6 cells [6]. Serological tests based on SARS-CoV-2 antibody detection, have been reported with varying sensitivity (34 to 80%), cross reactivity with other SARS-CoV, varying rates of seroconversion between 7 and 11 days after onset of symptoms and varying immunological responses [7,8]. Antigen detection assays also have the limitation of poor sensitivity and negative predictive values [7].

Therefore, rRT-PCR is the recommended reference test to establish laboratory diagnosis of SARS-CoV-2 by detecting at least two genes from various conserved region of specific structural Spike (*S*), Envelope (*E*), Nucleocapsid (*N*) genes and the nonstructural RNA dependent RNA polymerase (*RdRp*) and replicase open reading frame (*ORF*) 1a /b, *ORF 1b*-nsp14 [5,7,9]. Various in-house and commercially available rRT-PCR test kits are presently being used for identification of SARS-CoV-2 in the clinical specimens. OPS and/or NPS are currently the most preferred clinical specimens due to non-invasive and easily accessible nature and is being utilized across the globe to diagnose COVID-19 infection. During initial period of the pandemic in Wuhan, NPS was used to detect SARS-CoV-2 [5]. Since then, various studies, systemic reviews and meta-analysis have evaluated the spectrum of clinical specimens in the quest of optimal specimen for its inclusion in guidelines for early identification of SARS-CoV-2 to provide timely treatment to prevent its transmission and thus better management of the pandemic [5, 10-18]. These include upper respiratory tract specimen (saliva, OPS, NPS, nasal swab), lower respiratory specimen {sputum, bronchoalveolar lavage (BAL), endotracheal aspirate (ET), fibrobronchoscope brush biopsy (FBB)}, blood and its products (serum, plasma), urine, feces and rectal swab. These studies and meta-analysis have various conclusions, probably because of analyzing a different spectrum of clinical specimens. Systemic review and meta-analysis by Bwire et al. [17] and study by Wang W et al. [14] reported the highest SARS-CoV-2 detection rate in BAL, while

similar review and meta-analysis by Mohammadi et al. [18] and study by Zhang H et al. [13] recommends specimen of sputum for detection of SARS-CoV-2. Liu et al. [10] and Tong et al. [12] advocated NPS as specimen of choice for detection of nCoV. Rao et al. [11], on the other hand, found random saliva with a higher detection rate of nCoV than paired NPS and OPS swab. The optimal clinical specimens depend on various factors of ease of accessibility, non-invasive nature, a lesser risk to health care professional while collecting specimen and good viral loads for higher chances of detection. The collection of BAL, ET and FBB although have a higher detection rate and may be a specimen of choice in admitted pneumonia cases, yet it always poses a risk of generating aerosols to cause infection to healthcare workers. Additionally, they also cannot be a specimen of choice in managing pandemic infection of COVID-19 showing variable clinical manifestation from asymptomatic to mild/moderate and severe cases. Sputum, on the other hand, also pose a challenge not only for collection from cases of COVID-19 patients with dry cough but also for lower detection rate of nCoV as reported earlier [12]. Overall, there is certain uncertainty in understanding the specimens/sites from which the virus can be maximally diagnosed and which can be collected in field/community without posing health hazard to healthcare worker. Furthermore, these published studies have also not addressed optimal specimen in patients undergoing treatment to provide the appropriate prognostic indicator of viral clearance. Considering these facts, this study was undertaken to evaluate various clinical specimens that must be more accessible and feasible and can become a specimen of choice for early identification of SARS-CoV-2 for better management of COVID-19 pandemic. The proposed study has thus evaluated various specimens comprising of combined/paired naso and oropharyngeal swab (hereafter referred to as a combined swab in the text), NPS, OPS, sputum, plasma, serum, urine and ET from known positive COVID-19 patients to understand their diagnostic utility in detection of SARS-CoV-2 as well as monitoring of follow-up cases

of COVID-19 undergoing treatment. This study will also provide insight if this virus can also be transmitted in other ways than merely by respiratory droplets.

2. Methods

2.1. Patient selection

All India Institute of Medical Sciences (AIIMS)-Raipur is a designated tertiary-care hospital for diagnosis and treatment of COVID-19 patients in Chhattisgarh, a state in Central India. A total of 5000 suspected COVID-19 patients from May 2020 till June 2020, fulfilling either of the various testing criteria, laid down by the government of India, were referred to AIIMS, Raipur for diagnosis of COVID-19 infection by rRT-PCR test [19].

Among 5000-suspected patients, 137 outpatients were diagnosed for COVID-19 infection (2.7% positivity rate) by rRT-PCR using a combined swab. All these patients were subsequently admitted in the COVID ward of AIIMS, Raipur for isolation and treatment. These patients were evaluated in terms of the following inclusion and exclusion criteria.

Inclusion criteria

All suspected COVID-19 symptomatic patients were included in the study if fulfilling the following criteria-

a. Detected positive for COVID-19 infection by rRT-PCR.
b. Not on any anti-viral / anti-malarial (Hydroxychloroquine) / antibiotic (Azithromycin).
c. Admitted in COVID-19 ward of AIIMS, Raipur for treatment.

Exclusion criteria

a. Nonfulfillment of any of the inclusion criteria.

Among them, 07 patients with a recent history of taking Azithromycin were excluded. Accordingly, only 130 patients were enrolled in the study after taking their consent. This

study was approved by the Institutional ethical committee (IEC) of AIIMS, Raipur, Chhattisgarh (AIIMSRPR/IEC/2020/536).

Before starting a standard treatment regimen of Hydroxychloroquine and Azithromycin, all these patients were requested to provide clinical specimens of the following nature.

a. NPS
b. OPS
c. Combined (naso and oropharyngeal) swab
d. Sputum
e. Serum
f. Plasma
g. Urine

All swab specimens were collected from these patients before washing in morning by using sterile nylon flocked swab in viral transport medium (VTM) (HiMedia, India). An NPS was collected from a single nostril (posterior nasopharyngx) while OPS was collected from both sides of the throat. The combined swab of both NPS and OPS was collected in a single tube of VTM. In total, 910 (7 specimen types X 130 cases) specimens were tested by rRT-PCR. In addition, 14 ET were also obtained from an equal number of intubated patients. Thus, a total of 924 specimens were obtained from new patients prior to starting their treatment.

The positivity rate with all the seven types of clinical specimen was also tested in randomly selected 15 patients in their daily follow-up until the negative finding of rRT-PCR was achieved in two consecutive days' specimens of all seven types. Six hundred and sixty-five (665) follow-up specimens were collected from these 15 admitted patients. Thus, 924 initial and 665 follow-up specimens were tested by rRT-PCR for the identification of SARS-CoV-2.

2.2. RNA extraction

All the clinical specimens were processed for viral RNA isolation by using a commercially available QIAgen Viral RNA extraction kit, Germany, as per the manufacturer instructions. Briefly, 140µl of the specimen has been treated with 560µl of prepared buffer AVL containing carrier RNA (1 µg/µl). After brief pulse vortexing and 10-minutes incubation at room temperature, the specimen was precipitated by adding 560µl of pre-chilled ethanol. The treated specimen was then transferred to the spin column. Viral RNA was purified by consecutive treatment with 500µl of buffer AW1 and AW2. Finally, it was eluted in 60µl buffer AVE.

2.3. rRT-PCR test

This test was performed with primers and probes provided by Indian Council of Medical Research (ICMR), targeting *E*, *RdRP* and *ORF1b* genomic region of SARS-CoV-2 and internal control of human *RNAseP* as described earlier [20-22] (Table 1). Briefly, the 25 µl rRT-PCR reaction contained 12.5 µl 2x buffer, 1µl 25X RT-PCR enzyme mix (both from AgPath One-Step RT-PCR kit, ThermoFisher Scientific, USA), 1.5 µl Primer-Probe mix, 5 µl RNAse/DNase free sterile water and 5µl RNA template. The rRT-PCR test was carried out in CFX 96 Real Time PCR machine of Biorad, USA using the thermal cycling condition of 55^0C for 30 min, 95^0C for 3 min and 45 repeated cycles of 95^0C for 15 sec and 58^0C for 30 sec. The tested specimen was considered positive for SARS-CoV-2 for the cycle threshold (Ct) value less than or equal to 35 for *E* gene and both *RdRP* and *ORF1b* or either of *RdRP* or *ORF1b*. The positive and negative controls consisted of viral RNA plasmid and sterile nuclease-free water, respectively.

Table 1. Primer sequence of various genes of SARS-CoV-2 for rRT-PCR.

**Target gene**	**Sequence(5’-3’)**	**Source**
**E gene**	ACAGGTACGTTAATAGTTAATAGCGT	Corman et al. [20]
	ATATTGCAGCAGTACGCACACA	
	FAM-ACACTAGCCATCCTTACTGCGCTTCG-BHQ	
***RNaseP* (Internal**	AGATTTGGACCTGCGAGCG	CDC, 2020. [21]

**Control)**	GAGCGGCTGTCTCCACAAGT	
	FAM-TTCTGACCTGAAGGCTCTGCGCG-BHQ	
***RdRp* (Confirmatory)**	GTGARATGGTCATGTGTGGCGG	Corman et al. [20]
	CARATGTTAAASACACTATTAGCATA	
	FAM-CAGGTGGAACCTCATCAGGAGATGC-QSY	
***ORF1b* (Confirmatory)**	TGGGGYTTTACRGGTAACCT	Poon et al. [22]
	AACRCGCTTAACAAAGCACTC	
	FAM-TAGTTGTGATGCWATCATGACTAG-QSY	

2.4. Gold standard

All 130 rRT-PCR detected cases of COVID-19 infection were considered as the known positive cases to evaluate the efficacy of various clinical specimens for diagnostic utility.

2.5. Statistical analysis

Categorical variables were analyzed by chi-square (χ^2) and student t-test by using SPSS 16 version 18(SPSS Inc., Chicago, IL, USA) to compare intergroup detection rate by considering $p<0.05$ statistically significant.

3. Results

A total of 130 known positive cases of COVID-19 infection were evaluated in their 924 clinical specimens obtained from different anatomical sites by rRT-PCR to detect SARS-CoV-2. Demographic analysis of these patients showed the median age of 40.14 years (range 5 to 74 years). Among them, 86 were males while 44 were females showing a significant higher COVID-19 infection rate in males than females ($\chi^2 = 27.13$, $p=0.00001$, $p<0.05$). Median age calculated for males was 42.97 years, whereas, for females it was 32.07 years.

rRT-PCR detected all 130 cases with 100% positivity in combined swab (Table 2). NPS was the next appropriate clinical specimen showing a detection rate of 91.5%, followed by OPS and sputum specimens showing 72.3 and 63% positivity, respectively. None of the specimens of urine, plasma or serum showed detection of SARS-CoV-2. The 14 ET specimens showed 92.8% positivity by rRT-PCR. Combined swabs showed a significantly higher detection rate

of SARS-CoV-2 in comparison to NPS, OPS and Sputum (χ^2 =75.46, p=<0.001, p<0.05). On comparison of various individual specimens with combined swabs, a significant difference was noticed in positivity rate between combined swab versus NPS (χ^2 =11.48, p=0.0007, p<0.05), combined swab versus OPS (χ^2 =12.68, P=<0.001, p<0.05) and combined swab versus sputum (χ^2=58.86 p=<0.001, p<0.05). NPS positive detection rate was also found to be significantly higher as compared to OPS and sputum specimen (χ^2 =16.23, p=0.000056, p<0.05; χ^2 =30.01, p,0.00001, p<0.05). However, OPS positive detection rate was not found significantly higher than sputum positivity (χ^2 =2.53, p=0.11, p>0.05).

Table 2. Positivity of rRT-PCR in different clinical samples in 130 known COVID-19 patients.

**Total Patient (n=130)**	**Combined swab (n=130) No.(%)CI**	**NPS (n=130) No.(%) CI**	**OPS (n=130) No.(%) CI**	**Sputum (n=130) No.(%) CI**	**Urine (n=130) No.(%) CI**	**Plasma (n=130) No.(%) CI**	**Serum (n=130) No.(%) CI**	**Tracheal Aspirate (n=14) No.(%) CI**
**Male (n=86)**	86(100) (95.8-100)	79(91.5) (83.9-96.6)	63 (72.3) (62.6-82.2)	54(62.7) (51.7-72.9)	0~~(0)~~	0	0	13(92.8) (66.1-99.8)
**Female(n=44)**	44(100) (91.9-100)	40(90.9) (78.3-97.4)	31(70.4) (54.8-83.2)	28(63.6) (47.8-77.6)	0~~(0)~~	0	0	NA
**Total**	130(100) (97.2-100)	119(91.5) (85.3-95.7)	94(72.3) (63.8-79.8)	82(63.0) (54.2-71.4)	0~~(0)~~	0	0	13(92.8) (66.1-99.8)

Tracheal aspirate was obtained from 14 male cases only. ~~n(number tested)~~, No. (Number), %(Percentage), CI(Confidence Interval), ~~NA(No samples were obtained)~~.

Among individual swabs, NPS showed a higher detection rate than OPS. A total of 25 cases (19.2%, 16 males, 9 females) were detected in NPS but undetected in OPS (Table 2). However, a total of 11 (8.4%) cases were missed with NPS alone as the specimen, while nCoV was not detectable in 48 (36.9%) sputum specimens. No case was exclusively detected in OPS or sputum.

The Ct (threshold cycle) values of *ORF1b*, *RdRP* and *E* gene were also compared between different clinical specimens (Fig. 1). The cluttering of the Ct values was seen due to

maximum Ct values falling between 20 and 32. The lowest Ct values 10.56, 10.14 and 12.26 of *E*, *ORF1b* and *RdRP* were obtained in combined swab followed by NPS, Sputum and OPS, respectively (Fig. 1). The average Ct value of *E*, *ORF* and *RdRP* were 25.75, 26.94 and 27.06 in the combined swabs followed by NP, sputum and OP swabs respectively (Fig. 2). The theoretical correlation of inverse relationship between Ct values and viral load imperatively indicates of higher viral load in specimen with low Ct and vise-versa. Thus, it can be inferred that maximum viral load was present in the combined swab, followed by NPS, sputum and OPS. The specimens of urine, serum and plasma did not show any sigmoidal amplification-based Ct values. The t- test comparison of average Ct value of all the targeted genes namely *E*, *ORF1b* and *RdRp* in various specimen categories showed a significant difference when the combined swab was compared individually with NPS (p=0.021, t=-2.315), OPS (p=0.0003, t=-3.66) and sputum (p=0.0027, t= -3.028).

In randomly selected 15 follow up patients' testing, all seven types of specimens of combined swab, NPS, OPS, sputum, serum, plasma and urine were tested every day till the two consecutive days' rRT-PCR showed negative results in each specimen type (Fig. 3-4, Table 3). In the 'follow-up' category, a total of 665 specimens were obtained from 4 to 10 days after admission, with an average of 6.66 days (Fig. 3). A gradual increase in Ct values of *ORF1b* from combined swab, NPS, OPS and sputum were noticed in daily testing indicating patients' affirmative response to treatment and virus clearance while other specimens of plasma, serum and urine showed no detection of SARS-CoV-2 (Fig. 4). The maximum longer duration of days for clearance of virus was observed in combined swab (Fig. 4, Table 3). The earliest clearance with maximum detection of ORF1b was seen in patient P3 in which combined swab and NPS showed the presence of virus for only two treatment days and P11 in which only combined swab showed the presence of virus for two treatment days. Patients 1, 2, 7, 9, 11, 12, 13, 14 and 15 exclusively showed a longer duration of detection of nCOV in

combined swab. Patient 10 shed virus in combined and NPS specimen for longest nine days, followed by P7, which showed nCoV detection in only combined swab for consecutive seven days. During treatment monitoring, the average days of rRT-PCR positivity were 4.5, 3.7, 3.4 and 3.6 from the combined swab, NPS, OPS and sputum, respectively.

Table 3. *ORF1b* positivity of various samples for a maximum number of days in daily monitoring of 15 follow up cases.

Patient No.	ORF1b positivity for maximum number of days during treatment			
	**Combined swab**	**NPS**	**OPS**	**Sputum**
**P1**	5	4	4	4
**P2**	4	3	2	3
**P3**	2	2	1	1
**P4**	4	3	3	3
**P5**	4	4	4	4
**P6**	4	4	3	3
**P7**	7	5	5	5
**P8**	3	3	3	3
**P9**	4	2	2	2
**P10**	9	9	8	9
**P11**	2	1	1	1
**P12**	5	4	4	4
**P13**	6	5	5	5
**P14**	5	4	4	4
**P15**	4	3	3	3
**Average days positivity**	4.5	3.7	3.4	3.6

4. Discussion

The discharge policy for COVID-19 cases emphasized negative rRT-PCR findings on two consecutive days of respiratory specimen after symptom resolves. To give specific and accurate negative results, every laboratory needs to rule out false-negative PCR result, which otherwise would lead to discharge of such patient, leading to a high probability of transmission in the community, especially the family members and other close contacts. The importance of appropriate sampling in helping the laboratory to diagnose the COVID-19 infection accurately cannot be overemphasized. An appropriate specimen is the foundation

stone for good laboratory test result and is one of the essential pre-analytical parameters for quality assurance. It is well-accepted fact that improper specimen is bound to generate an incorrect result. It is therefore said that '*garbage in will yield garbage out*'. The appropriate specimen must also be the optimal specimen in monitoring treatment/follow-up cases to help the clinician in management by taking evidence based decision on discharge. This study was thus conducted to analyze the most appropriate specimen for performing rRT-PCR to diagnose SARS-CoV-2 and monitor follow-up cases.

The present study showed differences in sensitivity of combined swab in comparison to NPS and OPS with which 8.2 and 19.2% positive cases were missed respectively. Thus, if tested alone, NPS and OPS may cause remarkable false-negative results that could lead to a discharge of these infected patients who are still shedding SARS-CoV-2 from their upper respiratory tract and may be a potential source for transmission of COVID-19 infection. We have compared various studies to assess their finding of clinical suitability of different biodiversified specimens (Table 4). In a study by Wang X et al. [23], it was observed that 73.1% of positive nasopharyngeal cases could not be detected with OPS. Our study exclusively noted that 19.2% of cases were detected by only combined swabs and were missed by other specimen types. The detection rate in sputum was significantly lower as compared to combined swab and individual NPS and OPS. Thus, sputum specimen alone for diagnosis of COVID-19 infection by rRT-PCR may not be recommended, as it missed 36.9% of cases in the present study. Our finding is also corroborated by earlier reported study showing a low positivity rate of 28.53% using sputum in detection of nCoV [12]. However, our finding of low positivity in sputum is in contrast to some of the earlier reported studies and meta-analysis. The systemic review and meta-analysis earlier had found sputum a better specimen than NPS and OPS [18,19]. A separate study by Zhang H et al. [13] too found a higher detection rate of 79.2% in sputum than 37.5% and 20.8% positivity in NPS and OPS,

respectively. Among sputum and OPS, Wang W et al. [14] found higher positivity with sputum, whereas Chan et al. [24] and Liu R et al. [25] did not find any significant difference in positivity between them. We further have the opinion of sputum being a non-ideal specimen in patients of COVID-19 infection with symptoms of dry cough and unable to produce sputum.

Table 4. Comparative evaluation of our finding with earlier studies.

Study	Nature	No. of Samples	BAL	Sputum	NPS	OPS	Both swab	Feces	Blood	Urine	Rectal/ Anal swab	Serum	Plasma	FBB	ET	Nasal	Random saliva
**Wang W et al. [14]**	**Cross sectional**	Tested	15	104	8	398	-	153	307	72	-	-	-	13	-	-	-
		Positive	14	75	5	126	-	44	3	0	-	-	-	6	-	-	-
**Wang X et al. [23]**	**Cross sectional**	Tested	-	-	353	353	353	-	-	-	-	-	-	-	-	-	-
		Positive	-	-	67	27	76	-	-	-	-	-	-	-	-	-	-
**Xu et al. [34]**	**Prospective**	Tested	-	-	49	-	-	-	-	-	49	-	-	-	-	-	-
		Positive	-	-	22	-	-	-	-	-	43	-	-	-	-	-	-
**Lo et al. [26]**	**Prospective**	Tested	-	1	84	-	-	79	-	49	-	-	-	-	-	-	-
		Positive	-	1	57	-	-	46	-	0	-	-	-	-	-	-	-
**Chan et al. [24]**	**Case series**	Tested	-	3	5	3	-	4	-	5	-	3	4	-	-	-	-
		Positive	-	2	4	2	-	0	-	0	-	1	0	-	-	-	-
**Chen et al. [33]**	**Retrospective**	Tested	-	206	167	-	-	64	-	-	-	-	-	-	-	-	-
		Positive	-	155	65	-	-	17	-	-	-	-	-	-	-	-	-
**Liu R et al. [25]**	**Cross sectional**	Tested	5	57	4818	-	-	-	-	-	-	-	-	-	-	-	-
		Positive	4	28	1843	-	-	-	-	-	-	-	-	-	-	-	-
**Xie et al. [28]**	**Cross sectional**	Tested	-	-	-	19	-	19	19	19	-	-	-	-	-	-	-
		Positive	-	-	-	9	-	8	0	0	-	-	-	-	-	-	-
**Liu M et al. [10]**	**Cross sectional**	Tested	-	-	47	47	-	-	-	-	47	-	-	-	-	47	-
		Positive	-	-	26	9	-	-	-	-	1	-	-	-	-	23	-
**Rao et al. [11]**	**Cross sectional**	Tested	-	-	-	-	562	-	-	-	-	-	-	-	-	-	562
		Positive	-	-	-	-	48	-	-	-	-	-	-	-	-	-	60
**Tong et al. [12]**	**Cross sectional**	Tested	15	382	463	39	-	262	40	135	98	-	-	-	-	-	-

		Positive	7	61	297	10	-	32	3	12	8	-	-	-	-	-	-
**Zhang H et al. [13]**	**Cross sectional**	Tested	-	97	97	97	-	-	-	-	-	-	-	-	14	-	-
		Positive#	-	79.2	37.5	20.8	-	-	-	-	-	-	-	-	13	-	-
**Our study**	**Cross sectional**	Tested	-	130	130	130	130	-	-	-	-	-	-	-	14	-	-
		Positive	-	82	119	94	130	-	-	-	-	-	-	-	13	-	-

*This study did not show number of specimens detected. # Figures represent percentage.

If only one swab is to be used for COVID-19 diagnosis, then NPS should be preferred over other specimens of OPS, sputum, serum, plasma and urine considering its higher detection rate of nCoV in our study. This preference is in line with the earlier finding of Tong et al. [12], who found a higher detection rate of nCoV in NPS than BAL, OPS, sputum, urine, blood, stool, anal swabs and corneal secretions. The findings of Tong et al. [12], Lo et al. [26], Wang X et al. [23], Wang W et al. [14] and meta-analysis by Czumbel et al. [27] also showed NPS a better specimen for detecting SARS-CoV-2. The higher positivity rate of NPS could be correlated to higher viral load in nasopharynx than other anatomical sites/specimens.

Our study did not detect SARS-CoV-2 in clinical specimens of serum, plasma and urine. Earlier reported study too not found nCoV in either blood or urine specimens [28]. Chan et al. [24], Lo et al. [26], Wang W et al. [14] and reported meta-analysis showed negative results in urine specimen [17]. In contrast, only Tong et al. [12] had detected nCoV in urine, albeit with a low positivity rate of 16.3%. Low positivity rate of 12.5%, 1% and 0.9% was also reported in blood specimen by Tong et al. [12], Bwire et al. [17] and Wang W et al. [14], respectively. Chan et al. [24] found only one positive among three tested serum specimens while there was no positivity detected in plasma specimen. The number of specimens tested by Chan et al. [24] is too low to draw any relevant conclusion. Thus, it is advocated to conduct more studies on larger cohort to evaluate the role of blood and its components in diagnosing SARS-CoV-2 by rRT-PCR and its potential role in transmitting the virus. Ours

and earlier published analysis for the absence of SARS-CoV-2 in urine showed that it is not shed from the urogenital system. Among the optimal specimen, earlier published meta-analysis found BAL with higher positive rate of detection (91.8%) of SARS-CoV-2 followed by rectal swabs (87.8%), sputum (68.1%), nasopharyngeal swab (45.5%), feces (32.8%), oropharyngeal swab (7.6%), and blood samples (1.0%) [17]. Another meta-analysis on respiratory samples found sputum with a significantly higher positive rate of detection of nCoV followed by NPS and OPS [18]. Tong et al. [12], on the other hand, found NPS having highest positive detection rate of nCoV among other specimen spectrum of BAL, NPS, OPS, sputum, urine, blood, stool, anal swab and corneal secretion (2.99%) [12]. Rao et al. [11], found saliva a better specimen than paired NPS+ OPS swab. Thus, it is inferred, that an ideal appropriate specimen varied in above-discussed studies. However, considering, the fact that more studies find NPS an ideal specimen in the identification of nCoV, our suggested combined swab would be the most appropriate specimen in the pandemic situation due to fulfilling the parameters of applicability in the variable clinical spectrum of the disease, easy accessibility in a larger group of patients, lesser risk hazard to health worker and higher detection rate than NPS.

The present study also showed a high positive rate of COVID-19 in males than females, as infected males were almost twice that of females. The various earlier studies and meta-analysis too observed a higher male susceptibility than females to COVID-19 [14, 23, 29]. The prominent possible factors included higher expression of angiotensin-converting enzyme -2 (ACE-2) attachment receptors in males than females, higher incidence of heart disease, high blood pressure in males, immunological differences driven by hormones and X chromosome and behavioral difference of increased personal habits of smoking and consuming alcohol etc. Higher susceptibility of males was further precipitated by the reported epidemiological observation that males have a more casual approach than females in

appropriate compliance to wearing face mask, performing hand hygiene and maintaing social distancing practices [30, 31].

In terms of correlating lower Ct value with high viral load, our study showed detection of high viral load in the combined swab than other specimens. The individual NPS had the lowest Ct values in comparison to other individual specimens. This finding has also been corroborated by Wang W et al. [14], and Zou et al. [32], who also found higher viral load in NPS than OPS.

Our study also exclusively assessed the most appropriate clinical specimen to monitor the COVID-19 patients' treatment during their follow-up. Combined swabs exhibited longer duration of detection of nCoV as it is the last specimen during treatment follow-up to become negative among all seven types of specimens tested. This finding indicates that the combined swabs were the most appropriate specimen to assess virus clearance among the follow-up patients and thus equip the clinician in patient management and discharge. Data search found one brief report on 22 patients showing that sputum and feces remain positive even after NPS turned negative [33]. Another study on ten pediatric COVID 19 patients by Xu et al. [34] showed that rRT-PCR of rectal swabs was persistently positive after their NPS had become negative.

Novelty of the present study lies in the finding of combined swabs as an ideal specimen in both diagnosis and monitoring of treatment follow-up of symptomatic patients to better assess virus clearance, which eventually helps in discharge of truly recovered patients. This finding has clinical implication as early negative results with other specimens in follow-up investigation can give pseudoimpression of virus clearance leading to the potential risk of transmission of the COVID-19 infection in case if such patients are discharged. Among the published literature, Rao et al. [11], although found lower sensitivity of paired NPS + OPS

swab versus saliva in asymptomatic patient, the difference of study group leaves a scope of further study involving both symptomatic and asymptomatic patients. Nevertheless, the probable reason for higher positivity using combined swab in our study than Rao et al. [11] could be the more viral load in symptomatic than in asymptomatic patients and strict adherence to sample collection in morning without nasal and throat wash.

Although stool and rectal/anal swab specimen were not tested in our study, few studies showing detection of nCoV in these specimens indicate them as a potential specimen for diagnosis [5, 10, 12, 14, 17, 23]. These findings suggest that nCoV resist the human gut acidic medium and could be transmitted through the fecal route. Presence of nCoV in stool is also substantiated by evidential presence of its receptors ACE 2 in enterocytes. Nevertheless, the correlation of this potential biological specimen for diagnosis and probability of the virus transmission through feco-oral route deserves further evaluation, since the virus viability in stool has not yet fully explored except Wang W et al. [14] reporting live nCoV from the stool specimen.

The limitation of present study is non-evaluation of some of the other potential specimens like BAL, FBB, saliva, stool and rectal swab. Obtaining BAL and FBB was avoided since their collection requires an invasive procedure that may pose high-risk aerosol exposure to health care workers. The feces and rectal/anal swab are also not primarily indicated considering the respiratory droplet being the commonest established transmission mode of nCoV. Clinical specimen of feces and rectal/anal swab cannot be considered an optimal specimen considering the limitation of difficulty in collection, transport and processing in comparison to respiratory specimens. Another specimen of saliva has a variable reported finding. Apart from Rao et al. [11] who found saliva a better specimen, earlier reported meta-analysis and review had found saliva to be of low sensitivity than NPS [27, 35]. Saliva has also not been recommended by either WHO or our regional authorities (ICMR) in their

interim guidance for detection of SARS-CoV-2 [19, 36]. Therefore, saliva was not included in our study. We also could not correlate Ct values of *ORF1b* and *RdRP* with clinical features or disease course because most of the patients' detailed clinical information was not available.

Thus, this study concludes that NPS and OPS alone may miss some SARS-CoV-2 positive cases and hence should not be used exclusively as the sole specimen for diagnosis. Clinical specimen of serum, plasma and urine also should not be used for detection of SARS-CoV-2 by rRT-PCR. This study strongly recommends combined swab as the preferred clinical specimen for detection of SARS-CoV-2 to establish diagnosis of COVID-19. The combined swab may also be considered the most appropriate specimen for monitoring of the follow-up cases to provide a better prognostic indicator of viral clearance during treatment. Therefore, the combined swab specimen has tremendous translational value for defining the recommendation in testing guidelines. Implementing the same globally will help manage and control the pandemic, as it is the need of the hour. Lower Ct in combined and NPS specimen also indicates towards the indirect evidence of posterior nasopharynx as the primary nCoV colonization site. Since blood, serum, plasma and urine were negative for the presence of nCoV in our study, the other transmission routes were not confirmed in the study and requires more studies with larger sample size for specific conclusive finding.

Conflict of Interest

The authors have declared that no competing interest exists.

Funding Source

AIIMS, Raipur, Chhattisgarh (AIIMS/Raipur/Admin/110).

Authors Contributions

Negi SS, Nagarkar NM, Bhargava A and Das P contributed to conception and design of the manuscript. Behra A was responsible for management of clinical specimen. Sharma K, Aggarwala P, Gandhi D, Mathias A, Sharma S, Singh P, Negi SS contributed in processing, testing and analysis of the specimens by rRT-PCR. Gaikwad U and Wankhede A searched the literature and helped in comparative analysis of our test results with earlier studies. Negi SS wrote manuscript. Negi SS and Gandhi D helped in statistical analysis. Bhargava A, Das P and Nagarkar NM critically reviewed the manuscript.

References

1. Gorbalenya AE, Baker SC, Baric RS, de Groot RJ, Drosten C, Gulyaeva AA, et al. Severe acute respiratory syndrome-related coronavirus: The species and its viruses – a statement of the Coronavirus Study Group. bioRxiv. 2020; 2020.02.07.937862. doi:10.1101/2020.02.07.937862

2. WHO. WHO Coronavirus Disease (COVID-19) Dashboard. In: https://covid19.who.int/ [Internet]. 2020 [cited 11 Nov 2020]. Available: https://covid19.who.int/

3. Bhandari S, Singh A, Sharma R, Rankawat G, Banerjee S, Gupta V, et al. Characteristics, Treatment Outcomes and Role of Hydroxychloroquine among 522 COVID-19 hospitalized patients in Jaipur City: An Epidemio-Clinical Study. J Assoc Physicians India. 2020;68: 13–19.

4. Wu Z, McGoogan JM. Characteristics of and Important Lessons From the Coronavirus Disease 2019 (COVID-19) Outbreak in China: Summary of a Report of 72 314 Cases From the Chinese Center for Disease Control and Prevention. JAMA. 2020;323: 1239–1242. doi:10.1001/jama.2020.2648

5. Wang D, Hu B, Hu C, Zhu F, Liu X, Zhang J, et al. Clinical Characteristics of 138 Hospitalized Patients With 2019 Novel Coronavirus–Infected Pneumonia in Wuhan, China. JAMA. 2020;323: 1061–1069. doi:10.1001/jama.2020.1585

6. Takayama K. In Vitro and Animal Models for SARS-CoV-2 research. Trends Pharmacol Sci. 2020/05/30. 2020;41: 513–517. doi:10.1016/j.tips.2020.05.005

7. Abduljalil J. Laboratory diagnosis of SARS-CoV-2: available approaches and limitations. New Microbes New Infect. 2020;36: 100713. doi:10.1016/j.nmni.2020.100713

8. Kontou PI, Braliou GG, Dimou NL, Nikolopoulos G, Bagos PG. Antibody Tests in Detecting SARS-CoV-2 Infection: A Meta-Analysis. Diagnostics (Basel, Switzerland). 2020;10: 319. doi:10.3390/diagnostics10050319

9. Gao Z. Efficient management of novel coronavirus pneumonia by efficient prevention and control in scientific manner. Zhonghua Jie He He Hu Xi Za Zhi. 2020;43: E001. doi:10.3760/cma.j.issn.1001-0939.2020.0001

10. Liu M, Li Q, Zhou J, Ai W, Zheng X, Zeng J, et al. Value of swab types and collection time on SARS-COV-2 detection using RT-PCR assay. J Virol Methods. 2020/09/16. 2020;286: 113974. doi:10.1016/j.jviromet.2020.113974

11. Rao M, Rashid FA, Sabri FSAH, Jamil NN, Seradja V, Abdullah NA, et al. COVID-19 screening test by using random oropharyngeal saliva. J Med Virol. 2021;n/a. doi:https://doi.org/10.1002/jmv.26773

12. Tong Y, Bao A, Chen H, Huang J, Lv Z, Feng L, et al. Necessity for detection of SARS-CoV-2 RNA in multiple types of specimens for the discharge of the patients with COVID-19. J Transl Med. 2020;18: 411. doi:10.1186/s12967-020-02580-w

13. Zhang H, Chen M, Zhang Y, Wen J, Wang Y, Wang L, et al. The Yield and Consistency of the Detection of SARS-CoV-2 in Multiple Respiratory Specimens. Open Forum Infect Dis. 2020;7. doi:10.1093/ofid/ofaa379

14. Wang W, Xu Y, Gao R, Lu R, Han K, Wu G, et al. Detection of SARS-CoV-2 in Different Types of Clinical Specimens. JAMA. 2020;323: 1843–1844. doi:10.1001/jama.2020.3786

15. Zhang W, Du R-H, Li B, Zheng X-S, Yang X-L, Hu B, et al. Molecular and serological investigation of 2019-nCoV infected patients: implication of multiple shedding routes. Emerg Microbes Infect. 2020;9: 386–389. doi:10.1080/22221751.2020.1729071

16. Pan Y, Zhang D, Yang P, Poon LLM, Wang Q. Viral load of SARS-CoV-2 in clinical samples. Lancet Infect Dis. 2020/02/24. 2020;20: 411–412. doi:10.1016/S1473-3099(20)30113-4

17. Bwire GM, Majigo M V, Njiro BJ, Mawazo A. Detection profile of SARS-CoV-2 using RT-PCR in different types of clinical specimens: A systematic review and meta-analysis. J Med Virol. 2021;93: 719–725. doi:https://doi.org/10.1002/jmv.26349

18. Mohammadi A, Esmaeilzadeh E, Li Y, Bosch RJ, Li JZ. SARS-CoV-2 detection in different respiratory sites: A systematic review and meta-analysis. EBioMedicine. 2020;59: 102903. doi:https://doi.org/10.1016/j.ebiom.2020.102903

19. ICMR. Indian Council of Medical Research (ICMR).Strategy for COVID-19 testing in India. Version 5. In: https: // www.icmr.gov.in / pdf / covid / strategy / Testing_Strategy_v5_ 18052020.pdf. [Internet]. 2020 [cited 18 May 2020]. Available: https: // www.icmr.gov.in / pdf / covid / strategy / Testing_Strategy_v5_

18052020.pdf.

20. Corman VM, Landt O, Kaiser M, Molenkamp R, Meijer A, Chu DK, et al. Detection of 2019 novel coronavirus (2019-nCoV) by real-time RT-PCR. Euro Surveill. 2020;25: 2000045. doi:10.2807/1560-7917.ES.2020.25.3.2000045

21. WHO. CDC protocol of realtime RTPCR for influenza A (H1N1). In: https: // www . who. Int / csr / resources / publications / swineflu / real time rtpcr/en/. [Internet]. 2020 [cited 22 Sep 2020]. Available: https: // www . who. Int / csr / resources / publications / swineflu / real time rtpcr/en/.

22. Poon L, Chu D, Peiris M. Detection of 2019 novel coronavirus(2019-nCoV) in suspected human cases by RT-PCR. School of Public Health, The University of Hong Kong, Hong Kong. In: https://www.who.int/docs/default-source/coronaviruse/peiris-protocol-16-1-20. [Internet]. 2020 [cited 22 Sep 2020]. Available: https://www.who.int/docs/default-source/coronaviruse/peiris-protocol-16-1-20.

23. Wang X, Tan L, Wang X, Liu W, Lu Y, Cheng L, et al. Comparison of nasopharyngeal and oropharyngeal swabs for SARS-CoV-2 detection in 353 patients received tests with both specimens simultaneously. Int J Infect Dis. 2020/04/18. 2020;94: 107–109. doi:10.1016/j.ijid.2020.04.023

24. Chan JF-W, Yuan S, Kok K-H, To KK-W, Chu H, Yang J, et al. A familial cluster of pneumonia associated with the 2019 novel coronavirus indicating person-to-person transmission: a study of a family cluster. Lancet. 2020;395: 514–523. doi:10.1016/S0140-6736(20)30154-9

25. Liu R, Han H, Liu F, Lv Z, Wu K, Liu Y, et al. Positive rate of RT-PCR detection of SARS-CoV-2 infection in 4880 cases from one hospital in Wuhan, China, from Jan to

Feb 2020. Clin Chim Acta. 2020/03/07. 2020;505: 172–175. doi:10.1016/j.cca.2020.03.009

26. Lo IL, Lio CF, Cheong HH, Lei CI, Cheong TH, Zhong X, et al. Evaluation of SARS-CoV-2 RNA shedding in clinical specimens and clinical characteristics of 10 patients with COVID-19 in Macau. Int J Biol Sci. 2020;16: 1698–1707. doi:10.7150/ijbs.45357

27. Czumbel LM, Kiss S, Farkas N, Mandel I, Hegyi A, Nagy Á, et al. Saliva as a Candidate for COVID-19 Diagnostic Testing: A Meta-Analysis. Front Med. 2020;7: 465. doi:10.3389/fmed.2020.00465

28. Xie C, Jiang L, Huang G, Pu H, Gong B, Lin H, et al. Comparison of different samples for 2019 novel coronavirus detection by nucleic acid amplification tests. Int J Infect Dis. 2020;93: 264–267. doi:https://doi.org/10.1016/j.ijid.2020.02.050

29. Wei X, Xiao Y-T, Wang J, Chen R, Zhang W, Yang Y, et al. Sex Differences in Severity and Mortality Among Patients With COVID-19: Evidence from Pooled Literature Analysis and Insights from Integrated Bioinformatic Analysis. 2020.

30. Bwire GM. Coronavirus: Why Men are More Vulnerable to Covid-19 Than Women? SN Compr Clin Med. 2020; 1–3. doi:10.1007/s42399-020-00341-w

31. Sharma G, Volgman AS, Michos ED. Sex Differences in Mortality From COVID-19 Pandemic: Are Men Vulnerable and Women Protected? JACC Case reports. 2020/05/04. 2020;2: 1407–1410. doi:10.1016/j.jaccas.2020.04.027

32. Zou L, Ruan F, Huang M, Liang L, Huang H, Hong Z, et al. SARS-CoV-2 Viral Load in Upper Respiratory Specimens of Infected Patients. N Engl J Med. 2020;382: 1177–1179. doi:10.1056/NEJMc2001737

33. Chen C, Gao G, Xu Y, Pu L, Wang Q, Wang L, et al. SARS-CoV-2-Positive Sputum and Feces After Conversion of Pharyngeal Samples in Patients With COVID-19. Ann Intern Med. 2020/03/30. 2020;172: 832–834. doi:10.7326/M20-0991

34. Xu Y, Li X, Zhu B, Liang H, Fang C, Gong Y, et al. Characteristics of pediatric SARS-CoV-2 infection and potential evidence for persistent fecal viral shedding. Nat Med. 2020/03/13. 2020;26: 502–505. doi:10.1038/s41591-020-0817-4

35. Martinez RM. Clinical Samples for SARS-CoV-2 Detection: Review of the Early Literature. Clin Microbiol Newsl. 2020;42: 121–127. doi:https://doi.org/10.1016/j.clinmicnews.2020.07.001

36. WHO. Interim Guidance Diagnostic testing for SARS-CoV-2. In: https://apps.who.int/iris/bitstream/handle/10665/334254/WHO-2019-nCoV-laboratory-2020.6-eng [Internet]. 2020 [cited 3 Feb 2020]. Available: https://apps.who.int/iris/bitstream/handle/10665/334254/WHO-2019-nCoV-laboratory-2020.6-eng

Figure legends

Fig 1. The threshold cycle(Ct) values of *E*, *RdRP* and *ORF 1ab* region of SARS-CoV-2 in different clinical samples obtained from 130 patients. The lowest Ct values of all the three target of *E*, *RdRP* and *ORF 1ab* were obtained in combined Throat and nasopharyngeal swabs followed by NP, Sputum and Throat swabs. Urine, Plasma and Serum samples have not shown any amplification.

Fig 2. The average Ct value of *E*, *RdRP* and *ORF 1ab* gene in different clinical samples.

Fig 3. Number of samples tested for 15 follow up cases till rRT-PCR showed negative results in two consecutive days sample. Total number of samples per patients divided by 7 number of samples collected on daily basis gives the number of days the samples were collected for particular patients. Last two days 7 different types of samples were found negative for all the patients.

Fig 4. The values of Ct of *ORF 1ab* in various clinical samples of 15 follow up cases.

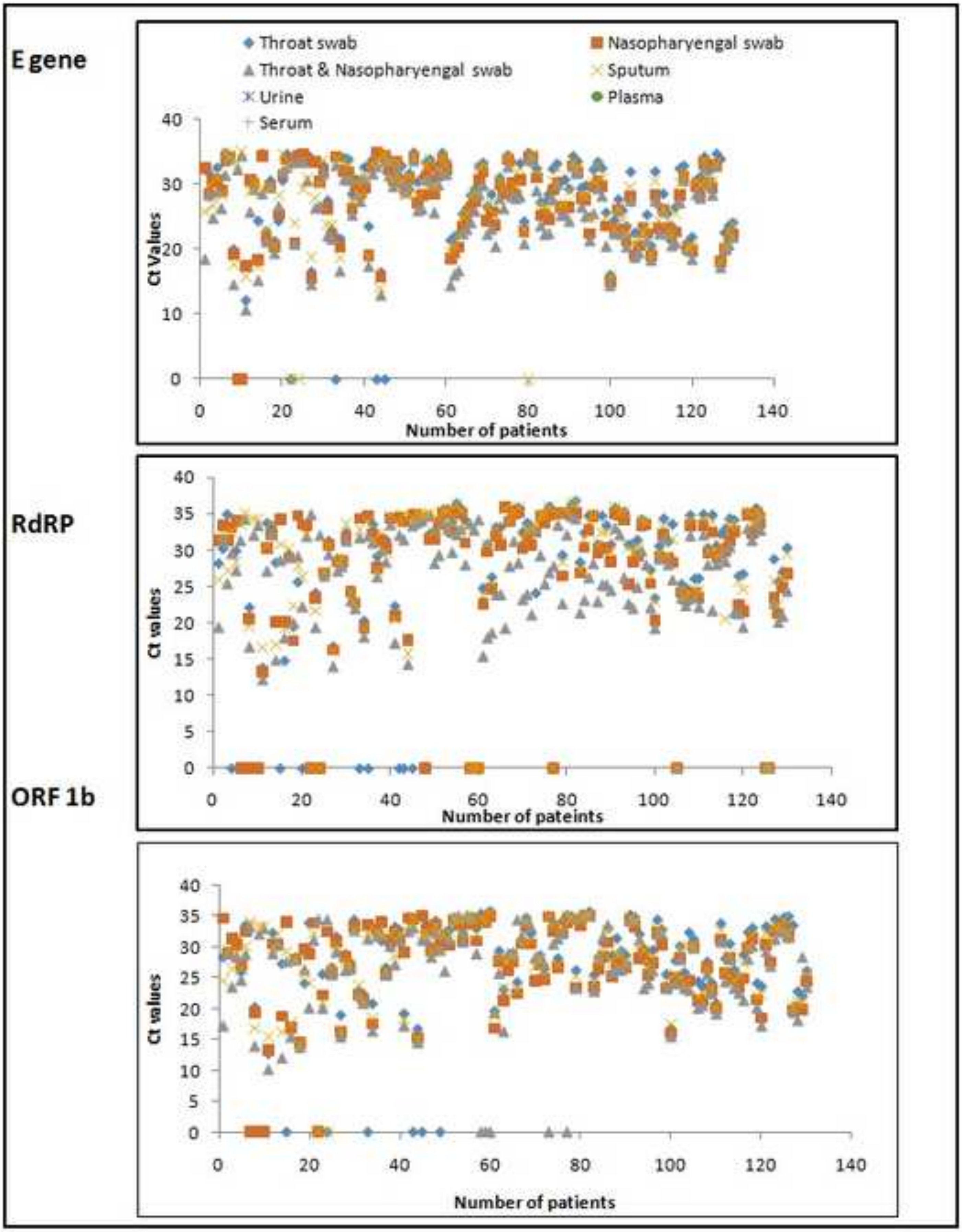
E gene
Throat swab
Nasopharyengal swab
Throat & Nasopharyengal swab
Sputum
Urine
Plasma
Serum
Ct Values
Number of patients
RdRP
Ct values
Number of pateints
ORF 1b
Ct values
Number of patients

Figure 2

Click here to access/download;Figure;Figure 2.jpg

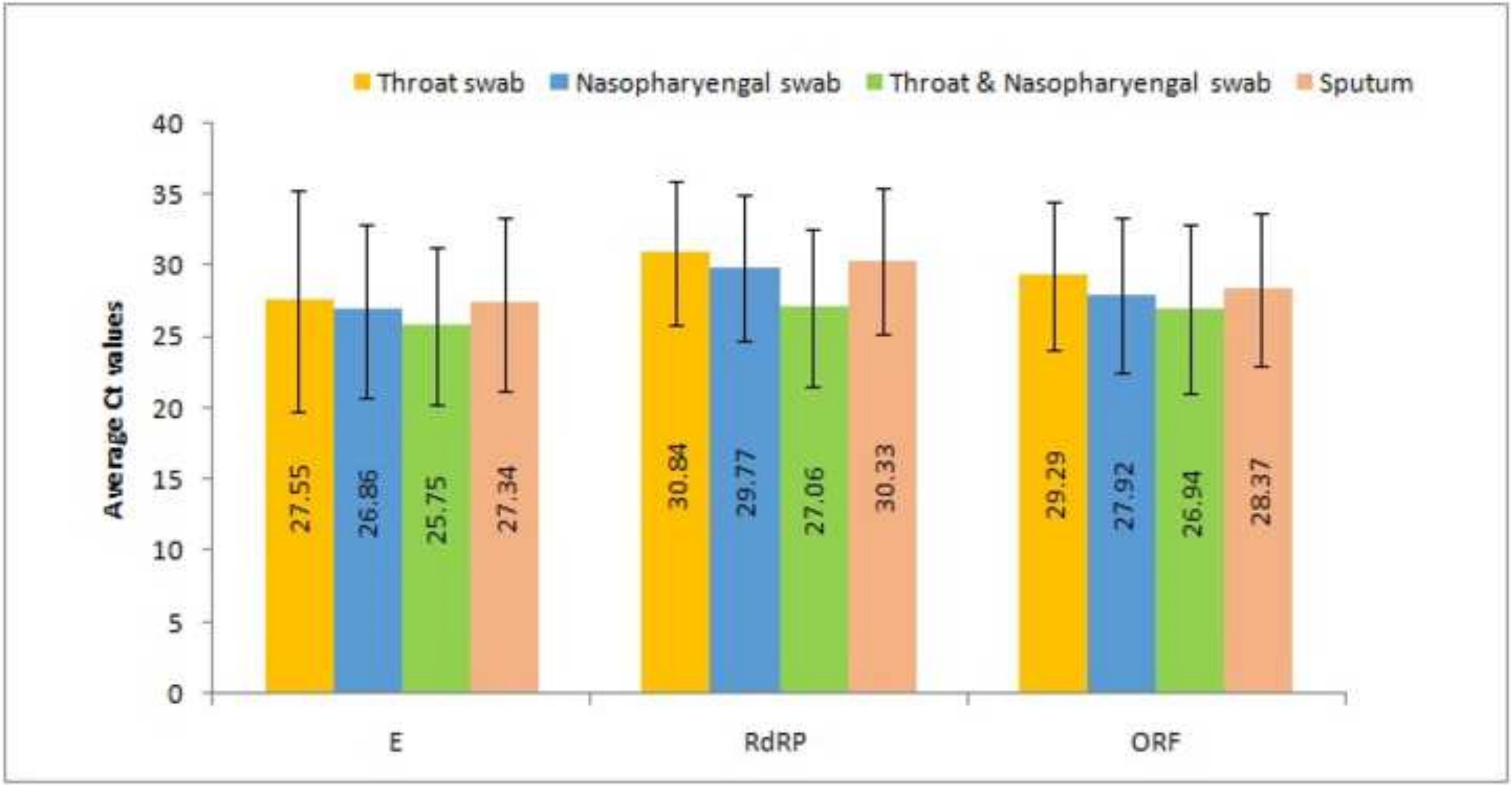

Figure 3

Click here to access/download;Figure;figure 3.jpg

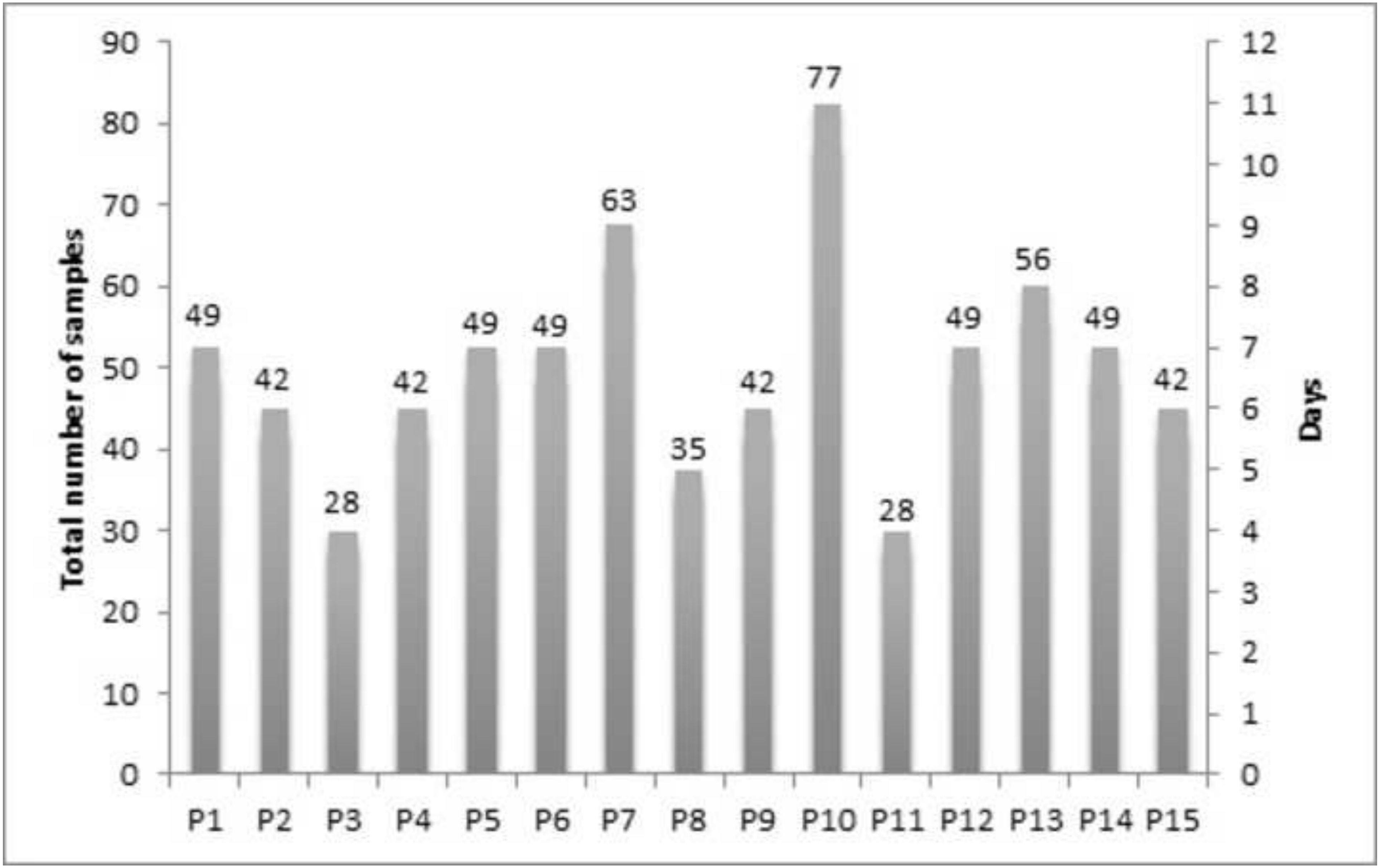

Figure 4

Click here to access/download;Figure;Figure 4 JPG revised 3.2.21.jpg

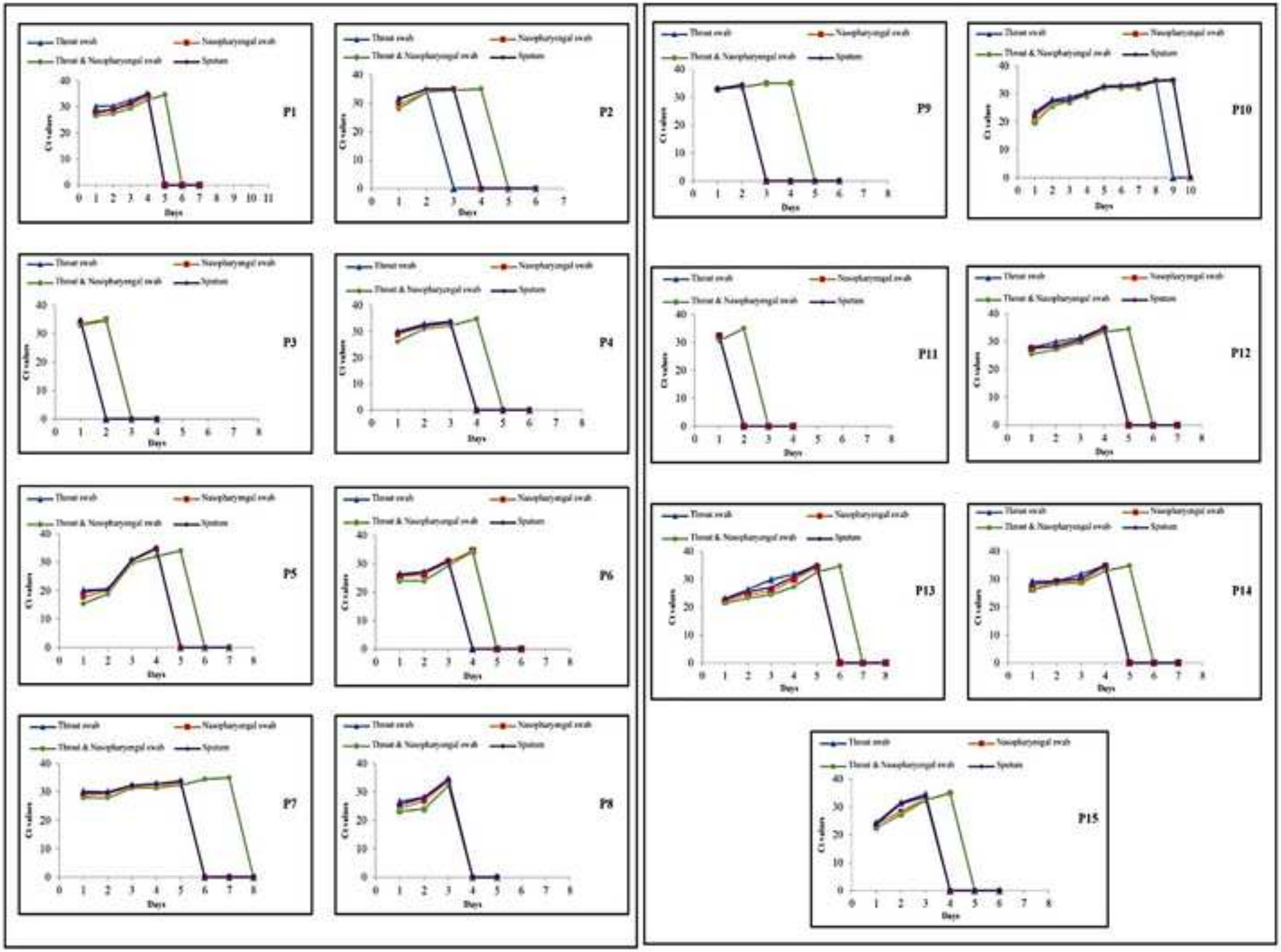

Comparative analysis of various clinical specimens in detection of SARS-CoV-2 using rRT-PCR in new and follow up cases of COVID-19 infection: quest for the best choice

Authors: Kuldeep Sharma[a,1], Pragya Aggarwala[a,1], Deepa Gandhi[a,1], Anuniti Mathias[a,1], Priyanka Singh[1], Saumya Sharma[1], Sanjay Singh Negi[1#], Anudita Bhargava[1], Padma Das[1], Ujjwala Gaikwad[1], Wankhede A[1], Behra A[2], Nitin M Nagarkar[3].

# Corresponding author

Dr. Sanjay Singh Negi
Associate Professor
Department of Microbiology
AIIMS, Raipur

[a] Kuldeep Sharma, Pragya Aggarwala, Deepa Gandhi, Anuniti Mathias contributed equally to this work as co-first authors.

[1] Microbiology Department, AIIMS, Raipur, Chhattisgarh.

[2] COVID Nodal Officer & Associate Professor, Department of Pulmonary Medicine, AIIMS, Raipur, Chhattisgarh.

[3] Director, AIIMS, Raipur, Chhattisgarh.

Running title: Evaluation of multivaried clinical specimens in diagnosis of COVID-19.

Abstract

An appropriate specimen is of paramount importance in Real Time reverse transcription-polymerase chain reaction (rRT-PCR) based diagnosis of novel coronavirus (nCoV) disease (COVID-19). Thus, it's pertinent to evaluate various diversified clinical specimens' diagnostic utility in both diagnosis and follow-up of COVID-19. A total of 924 initial specimens from 130 COVID-19 symptomatic cases before initiation of treatment and 665 follow up specimens from 15 randomly selected cases comprising of equal number of nasopharyngeal swab (NPS), oropharyngeal swab (OPS), combined NPS and OPS (Combined swab), sputum, plasma, serum and urine were evaluated by rRT-PCR. Combined swabs showed a positivity rate of 100 % followed by NPS (91.5%), OPS (72.3%), sputum (63%), while nCoV was found undetected in urine, plasma and serum specimens. The lowest cycle threshold (Ct) values of targeted genes *E, ORF1b* and *RdRP* of 10.56, 10.14 and 12.26 and their lowest average Ct values were found in combined swab which indicates high viral load in combined swab among all other specimen types. Analysis of 665 follow-up multi-varied specimens also showed combined swab as the last specimen among all specimen types to become negative, after an average 6.6 (range 4-10) days post-treatment, having lowest (15.48) and average(29.96) Ct values of *ORF1b* respectively indicating posterior nasopharyngeal tract as primary nCoV afflicted site with high viral load. The combined swab thus, may be recommendation as a more appropriate specimen for both diagnosis and monitoring of COVID-19 treatment by rRT-PCR for assessing virus clearance to help physicians in taking evidence-based decision before discharging patients. Implementing combined swabs globally will definitely help in management and control of the pandemic, as it is the need of the hour.

Key words: SARS-CoV-2, nCoV, rRT-PCR, combined swab.

Commented [DSN1]: Deleting "swab(NPS+OPS)" would lead to difficulty in understanding of the word 'combined'. Hence, rather than deleting those words, we have reframed them as "combined NPS and OPS".

Commented [DSN2]: The sentence has been reframed as suggested.

1. Introduction

SARS-CoV-2, a name given to a novel coronavirus (nCoV) by the International Committee of Taxonomy of Viruses (ICTV), was first reported in December 2019 from Wuhan, China. Since then, it has posed a devastating looming threat to the world, as around 216 countries and territories are so far affected by the virus causing the infection named COVID-19 [1]. Till the date of reporting on 11.11.2020, 50,810,763 were infected, and 1,263,844 succumbed worldwide to the infection [2]. India is the second most affected country after USA, with 8,636,011 confirmed cases and 127,571 deaths as of 11.11.2020 [2]. The disease can occur in any age-group, being more complicated and life-threatening in patients of the older age group and those with underlying co-morbid conditions such as diabetes, hypertension, cardiovascular and cerebrovascular disease [3-5]. The early clinical presentation of the COVID-19 varies from entirely asymptomatic to severely symptomatic. In India, more than 70% of the laboratory-confirmed cases are asymptomatic [3]. In symptomatic patients, the clinical manifestation includes fever, myalgia, dry cough, fatigue, productive cough, shortness of breath, chest pain, loss of taste and smell, etc. The radiological finding of ground-glass opacities on chest X-ray is one of the prominent observations [4,5]. Since SARS-CoV-2 has a high human-to-human transmissibility rate, early diagnosis, immediate isolation and early treatment of positive patients are key to successful management of the pandemic by preventing its spread to others. Since testing is the corner stone of managing the COVID-19 pandemic, highly sensitive and specific testing is essentially required for early identification of not only the symptomatic cases but also of the asymptomatic cases and their close high-risk contacts, which would potentially break the chain of transmission of COVID-19 infection, which otherwise appears unstoppable at the moment.

Among various viral diagnostic modalities, virus isolation does not appear practically feasible for COVID-19 since it requires Biosafety Level (BSL)-3 laboratory, high technical expertise

and longer turn-around time of 3-5 days to identify cytopathic effect in specific cell lines such as Vero E6 cells [6]. Serological tests based on SARS-CoV-2 antibody detection, have been reported with varying sensitivity (34 to 80%), cross reactivity with other SARS-CoV, varying rates of seroconversion between 7 and 11 days after onset of symptoms and varying immunological responses [7,8]. Antigen detection assays also have the limitation of poor sensitivity and negative predictive values [7].

Therefore, rRT-PCR is the recommended reference test to establish laboratory diagnosis of SARS-CoV-2 by detecting at least two genes from various conserved region of specific structural Spike (*S*), Envelope (*E*), Nucleocapsid (*N*) genes and the nonstructural RNA dependent RNA polymerase (*RdRp*) and replicase open reading frame (*ORF*) 1a /b, *ORF 1b*-nsp14 [5,7,9]. Various in-house and commercially available rRT-PCR test kits are presently being used for identification of SARS-CoV-2 in the clinical specimens. OPS and/or NPS are currently the most preferred clinical specimens due to non-invasive and easily accessible nature and is being utilized across the globe to diagnose COVID-19 infection. During initial period of the pandemic in Wuhan, NPS was used to detect SARS-CoV-2 [5]. Since then, various studies, systemic reviews and meta-analysis have evaluated the spectrum of clinical specimens in the quest of optimal specimen for its inclusion in guidelines for early identification of SARS-CoV-2 to provide timely treatment to prevent its transmission and thus better management of the pandemic [5, 10-18]. These include upper respiratory tract specimen (saliva, OPS, NPS, nasal swab), lower respiratory specimen {sputum, bronchoalveolar lavage (BAL), endotracheal aspirate (ET), fibrobronchoscope brush biopsy (FBB)}, blood and its products (serum, plasma), urine, feces and rectal swab. These studies and meta-analysis have various conclusions, probably because of analyzing a different spectrum of clinical specimens. Systemic review and meta-analysis by Bwire et al. [17] and study by Wang W et al. [14] reported the highest SARS-CoV-2 detection rate in BAL, while

Commented [DSN3]: The word 'by individual' has been changed as suggested.

Commented [DSN4]: The necessary changes has been incorporated in the revised manuscript as per the kind suggestion of the journal editorial team.

Commented [DSN5]: No its not a BAL. BAL specimen is mentioned in next line as bronchoalveolar lavage fluid (BLF).
For better clarity of the text, we have changed the abbreviation of BLF to BAL in the re-revised manuscript.

Commented [DSN6]: Space created between the words as suggested.

Commented [DSN7]: Space created between the words as suggested.

Commented [DSN8]: This has been already defined earlier in line number 72 as per the journal suggestion.

similar review and meta-analysis by Mohammadi et al. [18] and study by Zhang H et al. [13] recommends specimen of sputum for detection of SARS-CoV-2. Liu et al. [10] and Tong et al. [12] advocated NPS as specimen of choice for detection of nCoV. Rao et al. [11], on the other hand, found random saliva with a higher detection rate of nCoV than paired NPS and OPS swab. The optimal clinical specimens depend on various factors of ease of accessibility, non-invasive nature, a lesser risk to health care professional while collecting specimen and good viral loads for higher chances of detection. The collection of BAL, ET and FBB although have a higher detection rate and may be a specimen of choice in admitted pneumonia cases, yet it always poses a risk of generating aerosols to cause infection to healthcare workers. Additionally, they also cannot be a specimen of choice in managing pandemic infection of COVID-19 showing variable clinical manifestation from asymptomatic to mild/moderate and severe cases. Sputum, on the other hand, also pose a challenge not only for collection from cases of COVID-19 patients with dry cough but also for lower detection rate of nCoV as reported earlier [12]. Overall, there is certain uncertainty in understanding the specimens/sites from which the virus can be maximally diagnosed and which can be collected in field/community without posing health hazard to healthcare worker. Furthermore, these published studies have also not addressed optimal specimen in patients undergoing treatment to provide the appropriate prognostic indicator of viral clearance. Considering these facts, this study was undertaken to evaluate various clinical specimens that must be more accessible and feasible and can become a specimen of choice for early identification of SARS-CoV-2 for better management of COVID-19 pandemic. The proposed study has thus evaluated various specimens comprising of combined/paired naso and oropharyngeal swab (hereafter referred to as a combined swab in the text), NPS, OPS, sputum, plasma, serum, urine and ET from known positive COVID-19 patients to understand their diagnostic utility in detection of SARS-CoV-2 as well as monitoring of follow-up cases

of COVID-19 undergoing treatment. This study will also provide insight if this virus can also be transmitted in other ways than merely by respiratory droplets.

2. Methods

2.1. Patient selection

All India Institute of Medical Sciences (AIIMS)-Raipur is a designated tertiary-care hospital for diagnosis and treatment of COVID-19 patients in Chhattisgarh, a state in Central India. A total of 5000 suspected COVID-19 patients from May 2020 till June 2020, fulfilling either of the various testing criteria, laid down by the government of India, were referred to AIIMS, Raipur for diagnosis of COVID-19 infection by rRT-PCR test [19].

Among 5000-suspected patients, 137 outpatients were diagnosed for COVID-19 infection (2.7% positivity rate) by rRT-PCR using a combined swab. All these patients were subsequently admitted in the COVID ward of AIIMS, Raipur for isolation and treatment. These patients were evaluated in terms of the following inclusion and exclusion criteria.

Inclusion criteria

All suspected COVID-19 symptomatic patients were included in the study if fulfilling the following criteria-

a. Detected positive for COVID-19 infection by rRT-PCR.
b. Not on any anti-viral / anti-malarial (Hydroxychloroquine) / antibiotic (Azithromycin).
c. Admitted in COVID-19 ward of AIIMS, Raipur for treatment.

Exclusion criteria

a. Nonfulfillment of any of the inclusion criteria.

Among them, 07 patients with a recent history of taking Azithromycin were excluded. Accordingly, only 130 patients were enrolled in the study after taking their consent. This

study was approved by the Institutional ethical committee (IEC) of AIIMS, Raipur, Chhattisgarh (AIIMSRPR/IEC/2020/536).

Before starting a standard treatment regimen of Hydroxychloroquine and Azithromycin, all these patients were requested to provide clinical specimens of the following nature.

a. NPS
b. OPS
c. Combined (naso and oropharyngeal) swab
d. Sputum
e. Serum
f. Plasma
g. Urine

All swab specimens were collected from these patients before washing in morning by using sterile nylon flocked swab in viral transport medium (VTM) (HiMedia, India). An NPS was collected from a single nostril (posterior nasopharyngx) while OPS was collected from both sides of the throat. The combined swab of both NPS and OPS was collected in a single tube of VTM. In total, 910 (7 specimen types X 130 cases) specimens were tested by rRT-PCR. In addition, 14 ET were also obtained from an equal number of intubated patients. Thus, a total of 924 specimens were obtained from new patients prior to starting their treatment.

Commented [DSN9]: Yes, the 14 tracheal aspirate were obtained from 14 (equal number of) intubated patients. This needs to specify especially considering the fact that many a time, multiple tracheal aspirate are received from same group of patients.

The positivity rate with all the seven types of clinical specimen was also tested in randomly selected 15 patients in their daily follow-up until the negative finding of rRT-PCR was achieved in two consecutive days' specimens of all seven types. Six hundred and sixty-five (665) follow-up specimens were collected from these 15 admitted patients. Thus, 924 initial and 665 follow-up specimens were tested by rRT-PCR for the identification of SARS-CoV-2.

2.2. RNA extraction

All the clinical specimens were processed for viral RNA isolation by using a commercially available QIAgen Viral RNA extraction kit, Germany, as per the manufacturer instructions. Briefly, 140µl of the specimen has been treated with 560µl of prepared buffer AVL containing carrier RNA (1 µg/µl). After brief pulse vortexing and 10-minutes incubation at room temperature, the specimen was precipitated by adding 560µl of pre-chilled ethanol. The treated specimen was then transferred to the spin column. Viral RNA was purified by consecutive treatment with 500µl of buffer AW1 and AW2. Finally, it was eluted in 60µl buffer AVE.

2.3. rRT-PCR test

This test was performed with primers and probes provided by Indian Council of Medical Research (ICMR), targeting *E*, *RdRP* and *ORF1b* genomic region of SARS-CoV-2 and internal control of human *RNAseP* as described earlier [20-22] (Table 1). Briefly, the 25 µl rRT-PCR reaction contained 12.5 µl 2x buffer, 1µl 25X RT-PCR enzyme mix (both from AgPath One-Step RT-PCR kit, ThermoFisher Scientific, USA), 1.5 µl Primer-Probe mix, 5 µl RNAse/DNase free sterile water and 5µl RNA template. The rRT-PCR test was carried out in CFX 96 Real Time PCR machine of Biorad, USA using the thermal cycling condition of 55^0C for 30 min, 95^0C for 3 min and 45 repeated cycles of 95^0C for 15 sec and 58^0C for 30 sec. The tested specimen was considered positive for SARS-CoV-2 for the cycle threshold (Ct) value less than or equal to 35 for *E* gene and both *RdRP* and *ORF1b* or either of *RdRP* or *ORF1b*. The positive and negative controls consisted of viral RNA plasmid and sterile nuclease-free water, respectively.

Table 1. Primer sequence of various genes of SARS-CoV-2 for rRT-PCR.

Target gene	Sequence(5'-3')	Source
**E gene**	ACAGGTACGTTAATAGTTAATAGCGT	Corman et al. [20]
	ATATTGCAGCAGTACGCACACA	
	FAM-ACACTAGCCATCCTTACTGCGCTTCG-BHQ	
***RNaseP* (Internal**	AGATTTGGACCTGCGAGCG	CDC, 2020. [21]

**Control)**	GAGCGGCTGTCTCCACAAGT	
	FAM-TTCTGACCTGAAGGCTCTGCGCG-BHQ	
***RdRp* (Confirmatory)**	GTGARATGGTCATGTGTGGCGG	Corman et al. [20]
	CARATGTTAAASACACTATTAGCATA	
	FAM-CAGGTGGAACCTCATCAGGAGATGC-QSY	
***ORF1b* (Confirmatory)**	TGGGGYTTTACRGGTAACCT	Poon et al. [22]
	AACRCGCTTAACAAAGCACTC	
	FAM-TAGTTGTGATGCWATCATGACTAG-QSY	

2.4. Gold standard

All 130 rRT-PCR detected cases of COVID-19 infection were considered as the known positive cases to evaluate the efficacy of various clinical specimens for diagnostic utility.

2.5. Statistical analysis

Categorical variables were analyzed by chi-square (χ^2) and student t-test by using SPSS 16 version 18(SPSS Inc., Chicago, IL, USA) to compare intergroup detection rate by considering $p<0.05$ statistically significant.

3. Results

A total of 130 known positive cases of COVID-19 infection were evaluated in their 924 clinical specimens obtained from different anatomical sites by rRT-PCR to detect SARS-CoV-2. Demographic analysis of these patients showed the median age of 40.14 years (range 5 to 74 years). Among them, 86 were males while 44 were females showing a significant higher COVID-19 infection rate in males than females ($\chi^2 = 27.13$, $p=0.00001$, $p<0.05$). Median age calculated for males was 42.97 years, whereas, for females it was 32.07 years.

rRT-PCR detected all 130 cases with 100% positivity in combined swab (Table 2). NPS was the next appropriate clinical specimen showing a detection rate of 91.5%, followed by OPS and sputum specimens showing 72.3 and 63% positivity, respectively. None of the specimens of urine, plasma or serum showed detection of SARS-CoV-2. The 14 ET specimens showed 92.8% positivity by rRT-PCR. Combined swabs showed a significantly higher detection rate

of SARS-CoV-2 in comparison to NPS, OPS and Sputum (χ^2 =75.46, p=<0.001, p<0.05). On comparison of various individual specimens with combined swabs, a significant difference was noticed in positivity rate between combined swab versus NPS (χ^2 =11.48, p=0.0007, p<0.05), combined swab versus OPS (χ^2 =12.68, P=<0.001, p<0.05) and combined swab versus sputum (χ^2=58.86 p=<0.001, p<0.05). NPS positive detection rate was also found to be significantly higher as compared to OPS and sputum specimen (χ^2 =16.23, p=0.000056, p<0.05; χ^2 =30.01, p,0.00001, p<0.05). However, OPS positive detection rate was not found significantly higher than sputum positivity (χ^2 =2.53, p=0.11, p>0.05).

Table 2. Positivity of rRT-PCR in different clinical samples in 130 known COVID-19 patients.

**Total Patient (n=130)**	**Combined swab (n=130) No.(%)CI**	**NPS (n=130) No.(%) CI**	**OPS (n=130) No.(%) CI**	**Sputum (n=130) No.(%) CI**	**Urine (n=130) No.(%) CI**	**Plasma (n=130) No.(%) CI**	**Serum (n=130) No.(%) CI**	**Tracheal Aspirate (n=14) No.(%) CI**
**Male (n=86)**	86(100) (95.8-100)	79(91.5) (83.9-96.6)	63 (72.3) (62.6-82.2)	54(62.7) (51.7-72.9)	0(0)	0	0	13(92.8) (66.1-99.8)
**Female(n=44)**	44(100) (91.9-100)	40(90.9) (78.3-97.4)	31(70.4) (54.8-83.2)	28(63.6) (47.8-77.6)	0(0)	0	0	NA
**Total**	130(100) (97.2-100)	119(91.5) (85.3-95.7)	94(72.3) (63.8-79.8)	82(63.0) (54.2-71.4)	0(0)	0	0	13(92.8) (66.1-99.8)

Tracheal aspirate was obtained from 14 male cases only. n(number tested), No. (Number), %(Percentage), CI(Confidence Interval), NA(No samples were obtained).

Among individual swabs, NPS showed a higher detection rate than OPS. A total of 25 cases (19.2%, 16 males, 9 females) were detected in NPS but undetected in OPS (Table 2). However, a total of 11 (8.4%) cases were missed with NPS alone as the specimen, while nCoV was not detectable in 48 (36.9%) sputum specimens. No case was exclusively detected in OPS or sputum.

The Ct (threshold cycle) values of *ORF1b*, *RdRP* and *E* gene were also compared between different clinical specimens (Fig. 1). The cluttering of the Ct values was seen due to

maximum Ct values falling between 20 and 32. The lowest Ct values 10.56, 10.14 and 12.26 of *E*, *ORF1b* and *RdRP* were obtained in combined swab followed by NPS, Sputum and OPS, respectively (Fig. 1). The average Ct value of *E*, *ORF* and *RdRP* were 25.75, 26.94 and 27.06 in the combined swabs followed by NP, sputum and OP swabs respectively (Fig. 2). The theoretical correlation of inverse relationship between Ct values and viral load imperatively indicates of higher viral load in specimen with low Ct and vise-versa. Thus, it can be inferred that maximum viral load was present in the combined swab, followed by NPS, sputum and OPS. The specimens of urine, serum and plasma did not show any sigmoidal amplification-based Ct values. The t- test comparison of average Ct value of all the targeted genes namely *E*, *ORF1b* and *RdRp* in various specimen categories showed a significant difference when the combined swab was compared individually with NPS (p=0.021, t=-2.315), OPS (p=0.0003, t=-3.66) and sputum (p=0.0027, t= -3.028).

In randomly selected 15 follow up patients' testing, all seven types of specimens of combined swab, NPS, OPS, sputum, serum, plasma and urine were tested every day till the two consecutive days' rRT-PCR showed negative results in each specimen type (Fig. 3-4, Table 3). In the 'follow-up' category, a total of 665 specimens were obtained from 4 to 10 days after admission, with an average of 6.66 days (Fig. 3). A gradual increase in Ct values of *ORF1b* from combined swab, NPS, OPS and sputum were noticed in daily testing indicating patients' affirmative response to treatment and virus clearance while other specimens of plasma, serum and urine showed no detection of SARS-CoV-2 (Fig. 4). The maximum longer duration of days for clearance of virus was observed in combined swab (Fig. 4, Table 3). The earliest clearance with maximum detection of ORF1b was seen in patient P3 in which combined swab and NPS showed the presence of virus for only two treatment days and P11 in which only combined swab showed the presence of virus for two treatment days. Patients 1, 2, 7, 9, 11, 12, 13, 14 and 15 exclusively showed a longer duration of detection of nCOV in

combined swab. Patient 10 shed virus in combined and NPS specimen for longest nine days, followed by P7, which showed nCoV detection in only combined swab for consecutive seven days. During treatment monitoring, the average days of rRT-PCR positivity were 4.5, 3.7, 3.4 and 3.6 from the combined swab, NPS, OPS and sputum, respectively.

Table 3. *ORF1b* positivity of various samples for a maximum number of days in daily monitoring of 15 follow up cases.

Patient No.	ORF1b positivity for maximum number of days during treatment			
	**Combined swab**	**NPS**	**OPS**	**Sputum**
**P1**	5	4	4	4
**P2**	4	3	2	3
**P3**	2	2	1	1
**P4**	4	3	3	3
**P5**	4	4	4	4
**P6**	4	4	3	3
**P7**	7	5	5	5
**P8**	3	3	3	3
**P9**	4	2	2	2
**P10**	9	9	8	9
**P11**	2	1	1	1
**P12**	5	4	4	4
**P13**	6	5	5	5
**P14**	5	4	4	4
**P15**	4	3	3	3
**Average days positivity**	4.5	3.7	3.4	3.6

4. Discussion

The discharge policy for COVID-19 cases emphasized negative rRT-PCR findings on two consecutive days of respiratory specimen after symptom resolves. To give specific and accurate negative results, every laboratory needs to rule out false-negative PCR result, which otherwise would lead to discharge of such patient, leading to a high probability of transmission in the community, especially the family members and other close contacts. The importance of appropriate sampling in helping the laboratory to diagnose the COVID-19 infection accurately cannot be overemphasized. An appropriate specimen is the foundation

stone for good laboratory test result and is one of the essential pre-analytical parameters for quality assurance. It is well-accepted fact that improper specimen is bound to generate an incorrect result. It is therefore said that '*garbage in will yield garbage out*'. The appropriate specimen must also be the optimal specimen in monitoring treatment/follow-up cases to help the clinician in management by taking evidence based decision on discharge. This study was thus conducted to analyze the most appropriate specimen for performing rRT-PCR to diagnose SARS-CoV-2 and monitor follow-up cases.

The present study showed differences in sensitivity of combined swab in comparison to NPS and OPS with which 8.2 and 19.2% positive cases were missed respectively. Thus, if tested alone, NPS and OPS may cause remarkable false-negative results that could lead to a discharge of these infected patients who are still shedding SARS-CoV-2 from their upper respiratory tract and may be a potential source for transmission of COVID-19 infection. We have compared various studies to assess their finding of clinical suitability of different biodiversified specimens (Table 4). In a study by Wang X et al. [23], it was observed that 73.1% of positive nasopharyngeal cases could not be detected with OPS. Our study exclusively noted that 19.2% of cases were detected by only combined swabs and were missed by other specimen types. The detection rate in sputum was significantly lower as compared to combined swab and individual NPS and OPS. Thus, sputum specimen alone for diagnosis of COVID-19 infection by rRT-PCR may not be recommended, as it missed 36.9% of cases in the present study. Our finding is also corroborated by earlier reported study showing a low positivity rate of 28.53% using sputum in detection of nCoV [12]. However, our finding of low positivity in sputum is in contrast to some of the earlier reported studies and meta-analysis. The systemic review and meta-analysis earlier had found sputum a better specimen than NPS and OPS [18,19]. A separate study by Zhang H et al. [13] too found a higher detection rate of 79.2% in sputum than 37.5% and 20.8% positivity in NPS and OPS,

respectively. Among sputum and OPS, Wang W et al. [14] found higher positivity with sputum, whereas Chan et al. [24] and Liu R et al. [25] did not find any significant difference in positivity between them. We further have the opinion of sputum being a non-ideal specimen in patients of COVID-19 infection with symptoms of dry cough and unable to produce sputum.

Table 4. Comparative evaluation of our finding with earlier studies.

Study	Nature	No. of Samples	BAL	Sputum	NPS	OPS	Both swab	Feces	Blood	Urine	Rectal/ Anal swab	Serum	Plasma	FBB	ET	Nasal	Random saliva
**Wang W et al. [14]**	**Cross sectional**	Tested	15	104	8	398	-	153	307	72	-	-	-	13	-	-	-
		Positive	14	75	5	126	-	44	3	0	-	-	-	6	-	-	-
**Wang X et al. [23]**	**Cross sectional**	Tested	-	-	353	353	353	-	-	-	-	-	-	-	-	-	-
		Positive	-	-	67	27	76	-	-	-	-	-	-	-	-	-	-
**Xu et al. [34]**	**Prospective**	Tested	-	-	49	-	-	-	-	-	49	-	-	-	-	-	-
		Positive	-	-	22	-	-	-	-	-	43	-	-	-	-	-	-
**Lo et al. [26]**	**Prospective**	Tested	-	1	84	-	-	79	-	49	-	-	-	-	-	-	-
		Positive	-	1	57	-	-	46	-	0	-	-	-	-	-	-	-
**Chan et al. [24]**	**Case series**	Tested	-	3	5	3	-	4	-	5	-	3	4	-	-	-	-
		Positive	-	2	4	2	-	0	-	0	-	1	0	-	-	-	-
**Chen et al. [33]**	**Retrospectiv e**	Tested	-	206	167	-	-	64	-	-	-	-	-	-	-	-	-
		Positive	-	155	65	-	-	17	-	-	-	-	-	-	-	-	-
**Liu R et al. [25]**	**Cross sectional**	Tested	5	57	4818	-	-	-	-	-	-	-	-	-	-	-	-
		Positive	4	28	1843	-	-	-	-	-	-	-	-	-	-	-	-
**Xie et al. [28]**	**Cross sectional**	Tested	-	-	-	19	-	19	19	19	-	-	-	-	-	-	-
		Positive	-	-	-	9	-	8	0	0	-	-	-	-	-	-	-
**Liu M et al. [10]**	**Cross sectional**	Tested	-	-	47	47	-	-	-	-	47	-	-	-	-	47	-
		Positive	-	-	26	9	-	-	-	-	1	-	-	-	-	23	-
**Rao et al. [11]**	**Cross sectional**	Tested	-	-	-	-	562	-	-	-	-	-	-	-	-	-	562
		Positive	-	-	-	-	48	-	-	-	-	-	-	-	-	-	60
**Tong et al. [12]**	**Cross sectional**	Tested	15	382	463	39	-	262	40	13 5	98	-	-	-	-	-	-

		Positive	7	61	297	10	-	32	3	12	8	-	-	-	-	-	-
**Zhang H et al. [13]**	**Cross sectional**	Tested	-	97	97	97	-	-	-	-	-	-	-	-	14	-	-
		Positive#	-	79.2	37.5	20.8	-	-	-	-	-	-	-	-	13	-	-
**Our study**	**Cross sectional**	Tested	-	130	130	130	130	-	-	-	-	-	-	-	14	-	-
		Positive	-	82	119	94	130	-	-	-	-	-	-	-	13	-	-

*This study did not show number of specimens detected. # Figures represent percentage.

If only one swab is to be used for COVID-19 diagnosis, then NPS should be preferred over other specimens of OPS, sputum, serum, plasma and urine considering its higher detection rate of nCoV in our study. This preference is in line with the earlier finding of Tong et al. [12], who found a higher detection rate of nCoV in NPS than BAL, OPS, sputum, urine, blood, stool, anal swabs and corneal secretions. The findings of Tong et al. [12], Lo et al. [26], Wang X et al. [23], Wang W et al. [14] and meta-analysis by Czumbel et al. [27] also showed NPS a better specimen for detecting SARS-CoV-2. The higher positivity rate of NPS could be correlated to higher viral load in nasopharynx than other anatomical sites/specimens.

Our study did not detect SARS-CoV-2 in clinical specimens of serum, plasma and urine. Earlier reported study too not found nCoV in either blood or urine specimens [28]. Chan et al. [24], Lo et al. [26], Wang W et al. [14] and reported meta-analysis showed negative results in urine specimen [17]. In contrast, only Tong et al. [12] had detected nCoV in urine, albeit with a low positivity rate of 16.3%. Low positivity rate of 12.5%, 1% and 0.9% was also reported in blood specimen by Tong et al. [12], Bwire et al. [17] and Wang W et al. [14], respectively. Chan et al. [24] found only one positive among three tested serum specimens while there was no positivity detected in plasma specimen. The number of specimens tested by Chan et al. [24] is too low to draw any relevant conclusion. Thus, it is advocated to conduct more studies on larger cohort to evaluate the role of blood and its components in diagnosing SARS-CoV-2 by rRT-PCR and its potential role in transmitting the virus. Ours

and earlier published analysis for the absence of SARS-CoV-2 in urine showed that it is not shed from the urogenital system. Among the optimal specimen, earlier published meta-analysis found BAL with higher positive rate of detection (91.8%) of SARS-CoV-2 followed by rectal swabs (87.8%), sputum (68.1%), nasopharyngeal swab (45.5%), feces (32.8%), oropharyngeal swab (7.6%), and blood samples (1.0%) [17]. Another meta-analysis on respiratory samples found sputum with a significantly higher positive rate of detection of nCoV followed by NPS and OPS [18]. Tong et al. [12], on the other hand, found NPS having highest positive detection rate of nCoV among other specimen spectrum of BAL, NPS, OPS, sputum, urine, blood, stool, anal swab and corneal secretion (2.99%) [12]. Rao et al. [11], found saliva a better specimen than paired NPS+ OPS swab. Thus, it is inferred, that an ideal appropriate specimen varied in above-discussed studies. However, considering, the fact that more studies find NPS an ideal specimen in the identification of nCoV, our suggested combined swab would be the most appropriate specimen in the pandemic situation due to fulfilling the parameters of applicability in the variable clinical spectrum of the disease, easy accessibility in a larger group of patients, lesser risk hazard to health worker and higher detection rate than NPS.

The present study also showed a high positive rate of COVID-19 in males than females, as infected males were almost twice that of females. The various earlier studies and meta-analysis too observed a higher male susceptibility than females to COVID-19 [14, 23, 29]. The prominent possible factors included higher expression of angiotensin-converting enzyme -2 (ACE-2) attachment receptors in males than females, higher incidence of heart disease, high blood pressure in males, immunological differences driven by hormones and X chromosome and behavioral difference of increased personal habits of smoking and consuming alcohol etc. Higher susceptibility of males was further precipitated by the reported epidemiological observation that males have a more casual approach than females in

appropriate compliance to wearing face mask, performing hand hygiene and maintaing social distancing practices [30, 31].

In terms of correlating lower Ct value with high viral load, our study showed detection of high viral load in the combined swab than other specimens. The individual NPS had the lowest Ct values in comparison to other individual specimens. This finding has also been corroborated by Wang W et al. [14], and Zou et al. [32], who also found higher viral load in NPS than OPS.

Our study also exclusively assessed the most appropriate clinical specimen to monitor the COVID-19 patients' treatment during their follow-up. Combined swabs exhibited longer duration of detection of nCoV as it is the last specimen during treatment follow-up to become negative among all seven types of specimens tested. This finding indicates that the combined swabs were the most appropriate specimen to assess virus clearance among the follow-up patients and thus equip the clinician in patient management and discharge. Data search found one brief report on 22 patients showing that sputum and feces remain positive even after NPS turned negative [33]. Another study on ten pediatric COVID 19 patients by Xu et al. [34] showed that rRT-PCR of rectal swabs was persistently positive after their NPS had become negative.

Novelty of the present study lies in the finding of combined swabs as an ideal specimen in both diagnosis and monitoring of treatment follow-up of symptomatic patients to better assess virus clearance, which eventually helps in discharge of truly recovered patients. This finding has clinical implication as early negative results with other specimens in follow-up investigation can give pseudoimpression of virus clearance leading to the potential risk of transmission of the COVID-19 infection in case if such patients are discharged. Among the published literature, Rao et al. [11], although found lower sensitivity of paired NPS + OPS

swab versus saliva in asymptomatic patient, the difference of study group leaves a scope of further study involving both symptomatic and asymptomatic patients. Nevertheless, the probable reason for higher positivity using combined swab in our study than Rao et al. [11] could be the more viral load in symptomatic than in asymptomatic patients and strict adherence to sample collection in morning without nasal and throat wash.

Although stool and rectal/anal swab specimen were not tested in our study, few studies showing detection of nCoV in these specimens indicate them as a potential specimen for diagnosis [5, 10, 12, 14, 17, 23]. These findings suggest that nCoV resist the human gut acidic medium and could be transmitted through the fecal route. Presence of nCoV in stool is also substantiated by evidential presence of its receptors ACE 2 in enterocytes. Nevertheless, the correlation of this potential biological specimen for diagnosis and probability of the virus transmission through feco-oral route deserves further evaluation, since the virus viability in stool has not yet fully explored except Wang W et al. [14] reporting live nCoV from the stool specimen.

The limitation of present study is non-evaluation of some of the other potential specimens like BAL, FBB, saliva, stool and rectal swab. Obtaining BAL and FBB was avoided since their collection requires an invasive procedure that may pose high-risk aerosol exposure to health care workers. The feces and rectal/anal swab are also not primarily indicated considering the respiratory droplet being the commonest established transmission mode of nCoV. Clinical specimen of feces and rectal/anal swab cannot be considered an optimal specimen considering the limitation of difficulty in collection, transport and processing in comparison to respiratory specimens. Another specimen of saliva has a variable reported finding. Apart from Rao et al. [11] who found saliva a better specimen, earlier reported meta-analysis and review had found saliva to be of low sensitivity than NPS [27, 35]. Saliva has also not been recommended by either WHO or our regional authorities (ICMR) in their

interim guidance for detection of SARS-CoV-2 [19, 36]. Therefore, saliva was not included in our study. We also could not correlate Ct values of *ORF1b* and *RdRP* with clinical features or disease course because most of the patients' detailed clinical information was not available.

Thus, this study concludes that NPS and OPS alone may miss some SARS-CoV-2 positive cases and hence should not be used exclusively as the sole specimen for diagnosis. Clinical specimen of serum, plasma and urine also should not be used for detection of SARS-CoV-2 by rRT-PCR. This study strongly recommends combined swab as the preferred clinical specimen for detection of SARS-CoV-2 to establish diagnosis of COVID-19. The combined swab may also be considered the most appropriate specimen for monitoring of the follow-up cases to provide a better prognostic indicator of viral clearance during treatment. Therefore, the combined swab specimen has tremendous translational value for defining the recommendation in testing guidelines. Implementing the same globally will help manage and control the pandemic, as it is the need of the hour. Lower Ct in combined and NPS specimen also indicates towards the indirect evidence of posterior nasopharynx as the primary nCoV colonization site. Since blood, serum, plasma and urine were negative for the presence of nCoV in our study, the other transmission routes were not confirmed in the study and requires more studies with larger sample size for specific conclusive finding.

Conflict of Interest

The authors have declared that no competing interest exists.

Funding Source

AIIMS, Raipur, Chhattisgarh (AIIMS/Raipur/Admin/110).

Authors Contributions

Negi SS, Nagarkar NM, Bhargava A and Das P contributed to conception and design of the manuscript. Behra A was responsible for management of clinical specimen. Sharma K, Aggarwala P, Gandhi D, Mathias A, Sharma S, Singh P, Negi SS contributed in processing, testing and analysis of the specimens by rRT-PCR. Gaikwad U and Wankhede A searched the literature and helped in comparative analysis of our test results with earlier studies. Negi SS wrote manuscript. Negi SS and Gandhi D helped in statistical analysis. Bhargava A, Das P and Nagarkar NM critically reviewed the manuscript.

References

1. Gorbalenya AE, Baker SC, Baric RS, de Groot RJ, Drosten C, Gulyaeva AA, et al. Severe acute respiratory syndrome-related coronavirus: The species and its viruses – a statement of the Coronavirus Study Group. bioRxiv. 2020; 2020.02.07.937862. doi:10.1101/2020.02.07.937862

2. WHO. WHO Coronavirus Disease (COVID-19) Dashboard. In: https://covid19.who.int/ [Internet]. 2020 [cited 11 Nov 2020]. Available: https://covid19.who.int/

3. Bhandari S, Singh A, Sharma R, Rankawat G, Banerjee S, Gupta V, et al. Characteristics, Treatment Outcomes and Role of Hydroxychloroquine among 522 COVID-19 hospitalized patients in Jaipur City: An Epidemio-Clinical Study. J Assoc Physicians India. 2020;68: 13–19.

4. Wu Z, McGoogan JM. Characteristics of and Important Lessons From the Coronavirus Disease 2019 (COVID-19) Outbreak in China: Summary of a Report of 72 314 Cases From the Chinese Center for Disease Control and Prevention. JAMA. 2020;323: 1239–1242. doi:10.1001/jama.2020.2648

5. Wang D, Hu B, Hu C, Zhu F, Liu X, Zhang J, et al. Clinical Characteristics of 138 Hospitalized Patients With 2019 Novel Coronavirus–Infected Pneumonia in Wuhan, China. JAMA. 2020;323: 1061–1069. doi:10.1001/jama.2020.1585

6. Takayama K. In Vitro and Animal Models for SARS-CoV-2 research. Trends Pharmacol Sci. 2020/05/30. 2020;41: 513–517. doi:10.1016/j.tips.2020.05.005

7. Abduljalil J. Laboratory diagnosis of SARS-CoV-2: available approaches and limitations. New Microbes New Infect. 2020;36: 100713. doi:10.1016/j.nmni.2020.100713

8. Kontou PI, Braliou GG, Dimou NL, Nikolopoulos G, Bagos PG. Antibody Tests in Detecting SARS-CoV-2 Infection: A Meta-Analysis. Diagnostics (Basel, Switzerland). 2020;10: 319. doi:10.3390/diagnostics10050319

9. Gao Z. Efficient management of novel coronavirus pneumonia by efficient prevention and control in scientific manner. Zhonghua Jie He He Hu Xi Za Zhi. 2020;43: E001. doi:10.3760/cma.j.issn.1001-0939.2020.0001

10. Liu M, Li Q, Zhou J, Ai W, Zheng X, Zeng J, et al. Value of swab types and collection time on SARS-COV-2 detection using RT-PCR assay. J Virol Methods. 2020/09/16. 2020;286: 113974. doi:10.1016/j.jviromet.2020.113974

11. Rao M, Rashid FA, Sabri FSAH, Jamil NN, Seradja V, Abdullah NA, et al. COVID-19 screening test by using random oropharyngeal saliva. J Med Virol. 2021;n/a. doi:https://doi.org/10.1002/jmv.26773

12. Tong Y, Bao A, Chen H, Huang J, Lv Z, Feng L, et al. Necessity for detection of SARS-CoV-2 RNA in multiple types of specimens for the discharge of the patients with COVID-19. J Transl Med. 2020;18: 411. doi:10.1186/s12967-020-02580-w

13. Zhang H, Chen M, Zhang Y, Wen J, Wang Y, Wang L, et al. The Yield and Consistency of the Detection of SARS-CoV-2 in Multiple Respiratory Specimens. Open Forum Infect Dis. 2020;7. doi:10.1093/ofid/ofaa379

14. Wang W, Xu Y, Gao R, Lu R, Han K, Wu G, et al. Detection of SARS-CoV-2 in Different Types of Clinical Specimens. JAMA. 2020;323: 1843–1844. doi:10.1001/jama.2020.3786

15. Zhang W, Du R-H, Li B, Zheng X-S, Yang X-L, Hu B, et al. Molecular and serological investigation of 2019-nCoV infected patients: implication of multiple shedding routes. Emerg Microbes Infect. 2020;9: 386–389. doi:10.1080/22221751.2020.1729071

16. Pan Y, Zhang D, Yang P, Poon LLM, Wang Q. Viral load of SARS-CoV-2 in clinical samples. Lancet Infect Dis. 2020/02/24. 2020;20: 411–412. doi:10.1016/S1473-3099(20)30113-4

17. Bwire GM, Majigo M V, Njiro BJ, Mawazo A. Detection profile of SARS-CoV-2 using RT-PCR in different types of clinical specimens: A systematic review and meta-analysis. J Med Virol. 2021;93: 719–725. doi:https://doi.org/10.1002/jmv.26349

18. Mohammadi A, Esmaeilzadeh E, Li Y, Bosch RJ, Li JZ. SARS-CoV-2 detection in different respiratory sites: A systematic review and meta-analysis. EBioMedicine. 2020;59: 102903. doi:https://doi.org/10.1016/j.ebiom.2020.102903

19. ICMR. Indian Council of Medical Research (ICMR).Strategy for COVID-19 testing in India. Version 5. In: https: // www.icmr.gov.in / pdf / covid / strategy / Testing_Strategy_v5_ 18052020.pdf. [Internet]. 2020 [cited 18 May 2020]. Available: https: // www.icmr.gov.in / pdf / covid / strategy / Testing_Strategy_v5_

18052020.pdf.

20. Corman VM, Landt O, Kaiser M, Molenkamp R, Meijer A, Chu DK, et al. Detection of 2019 novel coronavirus (2019-nCoV) by real-time RT-PCR. Euro Surveill. 2020;25: 2000045. doi:10.2807/1560-7917.ES.2020.25.3.2000045

21. WHO. CDC protocol of realtime RTPCR for influenza A (H1N1). In: https: // www . who. Int / csr / resources / publications / swineflu / real time rtpcr/en/. [Internet]. 2020 [cited 22 Sep 2020]. Available: https: // www . who. Int / csr / resources / publications / swineflu / real time rtpcr/en/.

22. Poon L, Chu D, Peiris M. Detection of 2019 novel coronavirus(2019-nCoV) in suspected human cases by RT-PCR. School of Public Health, The University of Hong Kong, Hong Kong. In: https://www.who.int/docs/default-source/coronaviruse/peiris-protocol-16-1-20. [Internet]. 2020 [cited 22 Sep 2020]. Available: https://www.who.int/docs/default-source/coronaviruse/peiris-protocol-16-1-20.

23. Wang X, Tan L, Wang X, Liu W, Lu Y, Cheng L, et al. Comparison of nasopharyngeal and oropharyngeal swabs for SARS-CoV-2 detection in 353 patients received tests with both specimens simultaneously. Int J Infect Dis. 2020/04/18. 2020;94: 107–109. doi:10.1016/j.ijid.2020.04.023

24. Chan JF-W, Yuan S, Kok K-H, To KK-W, Chu H, Yang J, et al. A familial cluster of pneumonia associated with the 2019 novel coronavirus indicating person-to-person transmission: a study of a family cluster. Lancet. 2020;395: 514–523. doi:10.1016/S0140-6736(20)30154-9

25. Liu R, Han H, Liu F, Lv Z, Wu K, Liu Y, et al. Positive rate of RT-PCR detection of SARS-CoV-2 infection in 4880 cases from one hospital in Wuhan, China, from Jan to

Feb 2020. Clin Chim Acta. 2020/03/07. 2020;505: 172–175. doi:10.1016/j.cca.2020.03.009

26. Lo IL, Lio CF, Cheong HH, Lei CI, Cheong TH, Zhong X, et al. Evaluation of SARS-CoV-2 RNA shedding in clinical specimens and clinical characteristics of 10 patients with COVID-19 in Macau. Int J Biol Sci. 2020;16: 1698–1707. doi:10.7150/ijbs.45357

27. Czumbel LM, Kiss S, Farkas N, Mandel I, Hegyi A, Nagy Á, et al. Saliva as a Candidate for COVID-19 Diagnostic Testing: A Meta-Analysis. Front Med. 2020;7: 465. doi:10.3389/fmed.2020.00465

28. Xie C, Jiang L, Huang G, Pu H, Gong B, Lin H, et al. Comparison of different samples for 2019 novel coronavirus detection by nucleic acid amplification tests. Int J Infect Dis. 2020;93: 264–267. doi:https://doi.org/10.1016/j.ijid.2020.02.050

29. Wei X, Xiao Y-T, Wang J, Chen R, Zhang W, Yang Y, et al. Sex Differences in Severity and Mortality Among Patients With COVID-19: Evidence from Pooled Literature Analysis and Insights from Integrated Bioinformatic Analysis. 2020.

30. Bwire GM. Coronavirus: Why Men are More Vulnerable to Covid-19 Than Women? SN Compr Clin Med. 2020; 1–3. doi:10.1007/s42399-020-00341-w

31. Sharma G, Volgman AS, Michos ED. Sex Differences in Mortality From COVID-19 Pandemic: Are Men Vulnerable and Women Protected? JACC Case reports. 2020/05/04. 2020;2: 1407–1410. doi:10.1016/j.jaccas.2020.04.027

32. Zou L, Ruan F, Huang M, Liang L, Huang H, Hong Z, et al. SARS-CoV-2 Viral Load in Upper Respiratory Specimens of Infected Patients. N Engl J Med. 2020;382: 1177–1179. doi:10.1056/NEJMc2001737

33. Chen C, Gao G, Xu Y, Pu L, Wang Q, Wang L, et al. SARS-CoV-2-Positive Sputum and Feces After Conversion of Pharyngeal Samples in Patients With COVID-19. Ann Intern Med. 2020/03/30. 2020;172: 832–834. doi:10.7326/M20-0991

34. Xu Y, Li X, Zhu B, Liang H, Fang C, Gong Y, et al. Characteristics of pediatric SARS-CoV-2 infection and potential evidence for persistent fecal viral shedding. Nat Med. 2020/03/13. 2020;26: 502–505. doi:10.1038/s41591-020-0817-4

35. Martinez RM. Clinical Samples for SARS-CoV-2 Detection: Review of the Early Literature. Clin Microbiol Newsl. 2020;42: 121–127. doi:https://doi.org/10.1016/j.clinmicnews.2020.07.001

36. WHO. Interim Guidance Diagnostic testing for SARS-CoV-2. In: https://apps.who.int/iris/bitstream/handle/10665/334254/WHO-2019-nCoV-laboratory-2020.6-eng [Internet]. 2020 [cited 3 Feb 2020]. Available: https://apps.who.int/iris/bitstream/handle/10665/334254/WHO-2019-nCoV-laboratory-2020.6-eng

Comments and their reply

Comments 1: The format of the manuscript needs to be consistent, e.g. change Table 1 to (Table 1), Fig.1 to (Fig. 1), etc.

Reply: We sincerely apologize for our mistake. As per the suggestion, we have revised the format of the whole manuscript as per the PLOS One requirement.

Comment 2: Abstract, ling 8: Please spell out Please spell out NPS (nasopharyngeal swabs) and OPS (oropharyngeal swabs) here since this is the first time NPS and OPS were mentioned. What is combined swab? Do the authors mean combined nose throat swab? Please clarify.

Reply: As per the kind suggestion of the reviewer, we have spell out NPS and OPS as Nasopharyngeal and Oropharyngeal swab respectively in line 8 of the revised abstract. We further kindly submit that the combined swab consist of both nasopharyngeal and oropharyngeal swab in a single tube of viral transport medium. The same has been incorporated in the revised manuscript.

Comment 3: Line 33: "…in extreme of age.", Did the authors mean younger and older or just older? This needs rephrasing and clarify.

Reply: The authors meant to say that people of all ages are susceptible to COVID-19 infection. However, various studies have concluded beyond any doubt that older age group and persons with underlying conditions are more susceptible to COVID-19 infection and had a higher case fatality rate. As per the reviewer suggestion, we have reframed this line and it will now be read as

" The disease can occur in any age group being more complicated and life threatening in patients of older age group and those with underlying co- morbid conditions such as diabetes, hypertension, cardiovascular and cerebrovascular diseases (Bhandari S et al, 2020; Wu and McGoogan JM 2020; Wang D et al, 2020).

Comment 4: Line 69: Change "Sputum, Plasma, Serum, Urine and Tracheal aspirate" to "sputum, plasma, serum, urine and tracheal aspirate".

Reply: As per the suggestion, we have changed the "Sputum, Plasma, Serum, Urine and Tracheal aspirate" to "sputum, plasma, serum, urine and tracheal aspirate".

Comment 5: Line 130, change 2x buffer to 12.5 µl 2x buffer.
Line 97: Need approval #.

Reply: As per the suggestion, we have changed the 2x buffer to 12.5 µl 2x buffer in the revised manuscript.

We have obtained the approval from Institute Ethics Committee, AIIMS, Raipur, Chhattisgarh for the study. The ethical sanction number allotted is AIIMSRPR/IEC/2020/536. The same has been incorporated in the revised manuscript.

Comment 6: Line 137 – 139, 2.4 Gold standard: This is not how the gold standard is defined. I suggest deleting this section.

Reply: Respectfully, it is submitted that in the present study we have evaluated various clinical specimen for their diagnostic utility in detection of SARS-CoV-2 in both new and follow up cases. In such scenario, we need to ensure that the patients must be known diagnosed cases of COVID-19 infection. Since, real time PCR has been recommended throughout the world for diagnosis of SARS-CoV-2 in various clinical specimen during pandemic of COVID-19, we consider these 130 cases diagnosed by rRT-PCR as reference to analyze various clinical samples from them in initial and follow up stages.

Accordingly, we request you to kindly allow this to remain as such in the manuscript.

1. Comment 8: Line 266: The positive rate should be 0.9% not 0.009%.

Reply: We sincerely apologise for typological error. We have changed 0.009% to 0.9% in the revised manuscript.

Journal requirement

1. Please ensure that your manuscript meets PLOS ONE's style requirements, including those for file naming. The PLOS ONE style templates can be found at https://journals.plos.org/plosone/s/file?id=wjVg/PLOSOne_formatting_sample_main_body.pdf and https://journals.plos.org/plosone/s/file?id=ba62/PLOSOne_formatting_sample_title_authors_affiliations.pdf

   Our reply: We have ensured that our revised manuscript meet PLOS ONE’s style requirements.

2. We suggest you thoroughly copyedit your manuscript for language usage, spelling, and grammar.

   Our reply; We have done editing of our manuscript for language usage, spelling and grammar to best of our scientific knowledge.

3. Thank you for including the following ethics statement on the submission details page:
   'Study is approved form Institutional Ethical committee.
   Name of committe: IEC-AIIMS, Riapur.
   Approval number: AIIMSRPR/IEC/2020/536'
   Please also include the specific name of your ethics committee and the approval number in the Methods section of your manuscript."

Our reply: The specific name of the ethics committee is Institutional Ethical Committee(IEC), AIIMS, Raipur, Chhattisgarh. The same has been mentioned in the revised manuscript.

4. We note that you have indicated that data from this study are available upon request. PLOS only allows data to be available upon request if there are legal or ethical restrictions on sharing data publicly. For information on unacceptable data access restrictions, please see http://journals.plos.org/plosone/s/data-availability#loc-unacceptable-data-access-restrictions.

In your revised cover letter, please address the following prompts:

a) If there are ethical or legal restrictions on sharing a de-identified data set, please explain them in detail (e.g., data contain potentially identifying or sensitive patient information) and who has imposed them (e.g., an ethics committee). Please also provide contact information for a data access committee, ethics committee, or other institutional body to which data requests may be sent.

b) If there are no restrictions, please upload the minimal anonymized data set necessary to replicate your study findings as either Supporting Information files or to a stable, public repository and provide us with the relevant URLs, DOIs, or accession numbers. Please see http://www.bmj.com/content/340/bmj.c181.long for guidelines on how to de-identify and prepare clinical data for publication. For a list of acceptable repositories, please see http://journals.plos.org/plosone/s/data-availability#loc-recommended-repositories.

We will update your Data Availability statement on your behalf to reflect the information you provide.

Reply: We respectfully submit that data of the study contain the potentially identifying patient’s information. Therefore, these data most likely cannot be shared. However, any such request depends on permission from Institutional Ethical Committee, AIIMS, Raipur, Chhattisgarh.

We request the editor to update this statement on our behalf.

5. PLOS requires an ORCID iD for the corresponding author in Editorial Manager on papers submitted after December 6th, 2016. Please ensure that you have an ORCID iD and that it is validated in Editorial Manager. To do this, go to ‘Update my Information’ (in the upper left-hand corner of the main menu), and click on the Fetch/Validate link next to the ORCID field. This will take you to the ORCID site and allow you to create a new iD or authenticate a pre-existing iD in Editorial Manager. Please see the following

video for instructions on linking an ORCID iD to your Editorial Manager account: https://www.youtube.com/watch?v=_xcclfuvtxQ.

Reply: The ORCID iD for the corresponding author is 0000-0002-5292-9132 and the id is validated in Editorial Manager.

6. We note that you have included the phrase “data not shown” in your manuscript. Unfortunately, this does not meet our data sharing requirements. PLOS does not permit references to inaccessible data. We require that authors provide all relevant data within the paper, Supporting Information files, or in an acceptable, public repository. Please add a citation to support this phrase or upload the data that corresponds with these findings to a stable repository (such as Figshare or Dryad) and provide and URLs, DOIs, or accession numbers that may be used to access these data. Or, if the data are not a core part of the research being presented in your study, we ask that you remove the phrase that refers to these data.

Our reply: We kindly submit that NPS and OPS specimen detected 119 and 94 cases respectively leaving 25 cases undetected by OPS. This statement is adequately shown in Table 2. So we are removing the phrase “data not shown” and citing Table 2 in place of it.

7. Please include your tables as part of your main manuscript and remove the individual files. Please note that supplementary tables should be uploaded as separate "supporting information" files.

   Our reply: Agreeing with the suggestion, we have uploaded all the tables in the main revised manuscript.

Reviewer Comments:

1. It is clear when one looks at the cited works of the manuscript that the reference search of the authors ended in mid June 2020. No later paper is cited except a WHO guideline. This is unacceptable in this exploding area of research.

Both that introduction and the discussion must include recent comparative meta-analyses on the subject. In this respect, the Discussion should also emphasize the novelty of the present work compared to those.

Bwire GM, Majigo MV, Njiro BJ, Mawazo A. Detection profile of SARS-CoV-2 using RT-PCR in different types of clinical specimens: A systematic review and meta-analysis. J Med Virol. 2021 Feb;93(2):719-725. doi: 10.1002/jmv.26349. Epub 2020 Aug 2. PMID: 32706393; PMCID: PMC7404904.

Mohammadi A, Esmaeilzadeh E, Li Y, Bosch RJ, Li JZ. SARS-CoV-2 detection in different respiratory sites: A systematic review and meta-analysis. EBioMedicine. 2020 Sep;59:102903. doi: 10.1016/j.ebiom.2020.102903. Epub 2020 Jul 24. PMID: 32718896; PMCID: PMC7380223.

Our reply: It is kindly submitted that the authors had already mentioned and discussed the findings of all the research article included in the meta-analysis of Bwire et al like findings of Wang et al, Xu et al, Lo et al, Chan et al, Chen et al, Liu et al, Wang W et al. Further agreeing to your kind suggestion, we have included the specific finding of both these meta-analysis in our discussion. Novelty of the present work also has also been mentioned in revised manuscript mentioned as under.

Among all the published reports, novelty of the present study lies in its assessment of various clinical specimen in both diagnosis and follow-up of COVID-19 patients. To the best of authors' knowledge, none of the earlier studies evaluated combined swabs as the potential clinical specimen in both diagnosis and monitoring of treatment follow-up cases. Present study found combined swab to provide appropriate clinical picture of clearing of the viruses from the patient undergoing treatment as it was the last specimen among all tested specimen to turned negative. This finding has clinical implication as early negative result of other specimen in follow-up investigation can give pseudoimpression of virus clearance leading to potential risk of transmission of the COVID-19 infection in case if such patients are discharged.

Commented [DSN1]: Comment 1. It is clear when one looks at the cited works of the manuscript that the reference search of the authors ended in mid June 2020. No later paper is cited except a WHO guideline. This is unacceptable in this exploding area of research.

Both that introduction and the discussion must include recent comparative meta-analyses on the subject. In this respect, the Discussion should also emphasize the novelty of the present work compared to those.

2. Key original papers having similar aims as the present work should also be quoted and compared to the present data in the Discussion:

Liu M, Li Q, Zhou J, Ai W, Zheng X, Zeng J, Liu Y, Xiang X, Guo R, Li X, Wu X, Xu H, Jiang L, Zhang H, Chen J, Tian L, Luo J, Luo C. Value of swab types and collection time on SARS-COV-2 detection using RT-PCR assay. J Virol Methods. 2020 Dec;286:113974. doi: 10.1016/j.jviromet.2020.113974. Epub 2020 Sep 16. PMID: 32949663; PMCID: PMC7493793.

Tong Y, Bao A, Chen H, Huang J, Lv Z, Feng L, Cheng Y, Wang Y, Bai L, Rao W, Zheng H, Wu Z, Qiao B, Zhao Z, Wang H, Li Y. Necessity for detection of SARS-CoV-2 RNA in multiple types of specimens for the discharge of the patients with COVID-19. J Transl Med. 2020 Nov 2;18(1):411. doi: 10.1186/s12967-020-02580-w. PMID: 33138834; PMCID: PMC7605325.

Zhang H, Chen M, Zhang Y, Wen J, Wang Y, Wang L, Guo J, Liu C, Li D, Wang Y, Bai J, Gao G, Wang S, Yang D, Yu F, Yan L, Wan G, Zhang F. The Yield and Consistency of the Detection of SARS-CoV-2 in Multiple Respiratory Specimens. Open Forum Infect Dis. 2020 Aug 26;7(10):ofaa379. doi: 10.1093/ofid/ofaa379. PMID: 33072810; PMCID: PMC7499703.

Rao M, Rashid FA, Sabri FSAH, Jamil NN, Seradja V, Abdullah NA, Ahmad H, Aren SL, Ali SAS, Ghazali M, Manaf AA, Talib H, Hashim R, Zain R, Thayan R, Amran F, Aris T, Ahmad N. COVID-19 screening test by using random oropharyngeal saliva. J Med Virol. 2021 Jan 4. doi: 10.1002/jmv.26773. Epub ahead of print. PMID: 33393672.

Our reply: Sincerely acknowledging the kind comment of reviewer, we have incorporated all suggested studies and critically analyzed our result with finding of these studies. In doing so, we have re-framed the introduction and discussion section of the manuscript.

3. The authors suggested that their lower Ct in combined and NPS swab indicated the primary nCoV colonization site is the posterior nasopharynx. They did not provide any direct evidence for this. Therefor this should be removed from the major findings and conclusion. However, the indirect evidence that they provided should be discussed and compared to data of others received by other methodologies.

Our reply: As per the suggestion, we have removed our statement of primary nCoV colonization site is the posterior nasopharynx and modified it as indirect evidence in the discussion.

4. The data and the details of Figure 4 are simply invisible. Downloaded high resolution does not help on this a lot. For visibility and clarity, this figure should be completely redrawn.

Our reply: We have redrawn the figure 4. Hopefully it will be accessible in high resolution at your end.

5. The limitations of the study should be discussed in the Discussion in more details. For example, saliva is one of the most promising diagnostic sample. This should be discussed. At least the following meta-analysis should be cited and briefly credited in the discussion:

Czumbel LM, Kiss S, Farkas N, Mandel I, Hegyi A, Nagy Á, Lohinai Z, Szakács Z, Hegyi P, Steward MC, Varga G. Saliva as a Candidate for COVID-19 Diagnostic Testing: A Meta-Analysis. Front Med (Lausanne). 2020 Aug 4;7:465. doi: 10.3389/fmed.2020.00465. PMID: 32903849; PMCID: PMC7438940.

Our reply: Yes, the limitation of the study has been discussed in more detail in the discussion of revised manuscript.

6. The English language of the paper needs extensive revision by a professional language editor. Particularly, many sentences are very long, complicated, therefore, hard to understand.

Our reply: the English language is revised extensively in the revised manuscript.

6. PLOS authors have the option to publish the peer review history of their article (what does this mean?). If published, this will include your full peer review and any attached files.

Journal grammatical comments and our reply

General comment: While the manuscript is scientifically sound, there are format, typos and awkward sentences through out the manuscript that needs to be corrected (I have attached a file containing examples of my suggestion). We suggest you thoroughly copyedit your manuscript for language usage, spelling, and grammar.

Reply: As per the suggestion, we have point wise addressed all the suggestion shown in the attachment. The point wise reply is mentioned below. Moreover, we have also adequately addressed the issue of grammatical addressing of the manuscript. All reframed sentences in the manuscript has been highlighted in red in the revised manuscript.

Comments 1: Abstract, Line No. 9 is awkward sentence.

Reply: We have reframed the said sentence as "A total of 924 initial specimens from 130 COVID-19 symptomatic cases before initiation of treatment and 665 follow up specimens from 15 randomly selected cases comprising of equal number of nasopharyngeal swab (NPS), oropharyngeal swab (OPS), combined NPS and OPS (Combined swab), sputum, plasma, serum and urine were evaluated by rRT-PCR. Combined swabs showed a positivity rate of 100 % followed by NPS (91.5%), OPS (72.3%), sputum (63%), while nCoV was found undetected in urine, plasma and serum specimens" in the re-revised manuscript.

Comment 2: The lowest cycle threshold (Ct) values of 10.56, 10.14 and 12.26 and lowest average Ct values of targeted genes *E* ~~(25.75; CI 24.6-26.7), *ORF1b*(26.94; CI 25.9-27.9) and *RdRP*(27.06; CI 26.1-28)~~ were found in combined swab among all specimen types to indicate higher viral load in it.

Formatted: Justified

Reply: As per the suggestion, the necessary change has been incorporated in the re-revised manuscript.

Comment 3: Serological test based on SARS-CoV-2 antibody detection, have been reported with varying sensitivity (34 to 80%), cross reactivity with other SARS-CoV, varying rates of seroconversion between 7 and 11 days after onset of symptoms and varying immunological response by ~~individual s~~[7,8].

Reply: As per the suggestion, the necessary change has been incorporated in the re-revised manuscript.

Comment 4: These include upper respiratory tract specimen (saliva, OPS, NPS, nasal swab), lower respiratory specimen {sputum, bronchoalveolar lavage fluid (BLF) endotracheal aspirate(ET), fibrobronchoscope brush biopsy(FBB)}, blood and its products (serum, plasma), urine, feces and rectal swab.

Commented [DSN1]: Should this be BAL?

Reply: No its not a BAL. BAL specimen is mentioned in next line as bronchoalveolar lavage fluid (BLF). For better clarity of the text, we have changed the abbreviation of BLF to BAL in the re-revised manuscript.

The reframed sentence will be read as "These include upper respiratory tract specimen (saliva, OPS, NPS, nasal swab), lower respiratory specimen {sputum, bronchoalveolar lavage (BAL),

endotracheal aspirate (ET), fibrobronchoscope brush biopsy (FBB)}, blood and its products (serum, plasma), urine, feces and rectal swab."

Comment 5: Page no. 5, line no. 78, define BAL.

Reply: As suggested, we have defined BAL earlier in line number 71 of page number 4 in re-revised manuscript.

Comment 6: Page number 14, line number 144, " an equal number of what? male or females?

Reply: Yes, the 14 tracheal aspirate were obtained from 14 (equal number of) intubated patients. This needs to specify especially considering the fact that many a time, multiple tracheal aspirate are received from same group of patients.

Comment 7: Page number 25: Awkward sentence throughout the manuscript.

Reply: We have re-wrote majority of the sentences in the revised manuscript and all changes were highlighted in red.

Specific comment: While revising your submission, please upload your figure files to the Preflight Analysis and Conversion Engine (PACE) digital diagnostic tool, https://pacev2.apexcovantage.com/. PACE helps ensure that figures meet PLOS requirements. To use PACE, you must first register as a user. Registration is free. Then, login and navigate to the UPLOAD tab, where you will find detailed instructions on how to use the tool. If you encounter any issues or have any questions when using PACE, please email PLOS at figures@plos.org. Please note that Supporting Information files do not need this step.

Reply: As suggested, we have uploaded all four figures used in the manuscript.

Formatted: Font: (Default) Times New Roman, 12 pt
