# Supplementary material for: Comparative analysis of various clinical specimens in detection of SARS-CoV-2 using rRT-PCR in new and follow up cases of COVID-19 infection: Quest for the best choice
Source: PLoS One. 2021 Apr 5;16(4):e0249408. doi: 10.1371/journal.pone.0249408 (PMC8021168; doi:10.1371/journal.pone.0249408)
Supplement: Attachment — Submitted filename: Response to Reviewers.docx [file pone.0249408.s001.docx]

Comments and their reply

Comments 1: The format of the manuscript needs to be consistent, e.g. change Table 1 to (Table 1), Fig.1 to (Fig. 1), etc.

Reply: We sincerely apologize for our mistake. As per the suggestion, we have revised the format of the whole manuscript as per the PLOS One requirement.

Comment 2: Abstract, ling 8:  Please spell out Please spell out NPS (nasopharyngeal swabs) and OPS (oropharyngeal swabs) here since this is the first time NPS and OPS were mentioned. What is combined swab? Do the authors mean combined nose throat swab? Please clarify.

Reply: As per the kind suggestion of the reviewer, we have spell out NPS and OPS as Nasopharyngeal and Oropharyngeal swab respectively in line 8 of the revised abstract. We further kindly submit that the combined swab consist of both nasopharyngeal and oropharyngeal swab in a single tube of viral transport medium. The same has been incorporated in the revised manuscript.

Comment 3: Line 33: “…in extreme of age.”, Did the authors mean younger and older or just older? This needs rephrasing and clarify.

Reply: The authors meant to say that people of all ages are susceptible to COVID-19 infection. However, various studies have concluded beyond any doubt that older age group and persons with underlying conditions are more susceptible to COVID-19 infection and had a higher case fatality rate. As per the reviewer suggestion, we have reframed this line and it will now be read as

“ The disease can occur in any age group being more complicated and life threatening in patients of older age group and those with underlying co- morbid conditions such as diabetes, hypertension, cardiovascular and cerebrovascular diseases (Bhandari S et al, 2020; Wu and McGoogan JM 2020; Wang D et al, 2020).

Comment 4: Line 69:  Change “Sputum, Plasma, Serum, Urine and Tracheal aspirate” to “sputum, plasma, serum, urine and tracheal aspirate”.

Reply: As per the suggestion, we have changed the “Sputum, Plasma, Serum, Urine and Tracheal aspirate” to “sputum, plasma, serum, urine and tracheal aspirate”.

Comment 5: Line 130, change 2x buffer to 12.5 μl 2x buffer.

Line 97:  Need approval #.

Reply: As per the suggestion, we have changed the 2x buffer to 12.5 μl 2x buffer in the revised manuscript.

We have obtained the approval from Institute Ethics Committee, AIIMS, Raipur, Chhattisgarh for the study. The ethical sanction number allotted is AIIMSRPR/IEC/2020/536. The same has been incorporated in the revised manuscript.

Comment 6: Line 137 – 139, 2.4 Gold standard:  This is not how the gold standard is defined.  I suggest deleting this section.

Reply: Respectfully, it is submitted that in the present study we have evaluated various clinical specimen for their diagnostic utility in detection of SARS-CoV-2 in both new and follow up cases. In such scenario, we need to ensure that the patients must be known diagnosed cases of COVID-19 infection. Since, real time PCR has been recommended throughout the world for diagnosis of SARS-CoV-2 in various clinical specimen during pandemic of COVID-19, we consider these 130 cases diagnosed by rRT-PCR as reference to analyze various clinical samples from them in initial and follow up stages.

Accordingly, we request you to kindly allow this to remain as such in the manuscript.

1. Comment 8: Line 266:  The positive rate should be 0.9% not 0.009%.

Reply: We sincerely apologise for typological error. We have changed 0.009% to 0.9% in the revised manuscript.

Journal requirement

1. Please ensure that your manuscript meets PLOS ONE's style requirements, including those for file naming. The PLOS ONE style templates can be found at

https://journals.plos.org/plosone/s/file?id=wjVg/PLOSOne_formatting_sample_main_body.pdf and

https://journals.plos.org/plosone/s/file?id=ba62/PLOSOne_formatting_sample_title_authors_affiliations.pdf

Our reply: We have ensured that our revised manuscript meet PLOS ONE’s style requirements.

2. We suggest you thoroughly copyedit your manuscript for language usage, spelling, and grammar.

Our reply; We have done editing of our manuscript for language usage, spelling and grammar to best of our scientific knowledge.

3. Thank you for including the following ethics statement on the submission details page:
   'Study is approved form Institutional Ethical committee.
   Name of committe: IEC-AIIMS, Riapur.
   Approval number: AIIMSRPR/IEC/2020/536'

Please also include the specific name of your ethics committee and the approval number in the Methods section of your manuscript."

Our reply: The specific name of the ethics committee is Institutional Ethical Committee(IEC), AIIMS, Raipur, Chhattisgarh. The same has been mentioned in the revised manuscript.

4. We note that you have indicated that data from this study are available upon request. PLOS only allows data to be available upon request if there are legal or ethical restrictions on sharing data publicly. For information on unacceptable data access restrictions, please see http://journals.plos.org/plosone/s/data-availability#loc-unacceptable-data-access-restrictions.

In your revised cover letter, please address the following prompts:

 a) If there are ethical or legal restrictions on sharing a de-identified data set, please explain them in detail (e.g., data contain potentially identifying or sensitive patient information) and who has imposed them (e.g., an ethics committee). Please also provide contact information for a data access committee, ethics committee, or other institutional body to which data requests may be sent.

 b) If there are no restrictions, please upload the minimal anonymized data set necessary to replicate your study findings as either Supporting Information files or to a stable, public repository and provide us with the relevant URLs, DOIs, or accession numbers. Please see http://www.bmj.com/content/340/bmj.c181.long for guidelines on how to de-identify and prepare clinical data for publication. For a list of acceptable repositories, please see http://journals.plos.org/plosone/s/data-availability#loc-recommended-repositories.

 We will update your Data Availability statement on your behalf to reflect the information you provide.

Reply: We respectfully submit that data of the study contain the potentially identifying patient’s information. Therefore, these data most likely cannot be shared. However, any such request depends on permission from Institutional Ethical Committee, AIIMS, Raipur, Chhattisgarh.

We request the editor to update this statement on our behalf.

5. PLOS requires an ORCID iD for the corresponding author in Editorial Manager on papers submitted after December 6th, 2016. Please ensure that you have an ORCID iD and that it is validated in Editorial Manager. To do this, go to ‘Update my Information’ (in the upper left-hand corner of the main menu), and click on the Fetch/Validate link next to the ORCID field. This will take you to the ORCID site and allow you to create a new iD or authenticate a pre-existing iD in Editorial Manager. Please see the following video for instructions on linking an ORCID iD to your Editorial Manager account: <https://www.youtube.com/watch?v=_xcclfuvtxQ>.

Reply: The ORCID iD for the corresponding author is 0000-0002-5292-9132 and the id is validated in Editorial Manager.

6. We note that you have included the phrase “data not shown” in your manuscript. Unfortunately, this does not meet our data sharing requirements. PLOS does not permit references to inaccessible data. We require that authors provide all relevant data within the paper, Supporting Information files, or in an acceptable, public repository. Please add a citation to support this phrase or upload the data that corresponds with these findings to a stable repository (such as Figshare or Dryad) and provide and URLs, DOIs, or accession numbers that may be used to access these data. Or, if the data are not a core part of the research being presented in your study, we ask that you remove the phrase that refers to these data.

Our reply: We kindly submit that NPS and OPS specimen detected 119 and 94 cases respectively leaving 25 cases undetected by OPS. This statement is adequately shown in Table 2. So we are removing the phrase “data not shown” and citing Table 2 in place of it.

7. Please include your tables as part of your main manuscript and remove the individual files. Please note that supplementary tables should be uploaded as separate "supporting information" files.

Our reply: Agreeing with the suggestion, we have uploaded all the tables in the main revised manuscript.

Reviewer Comments:

1. It is clear when one looks at the cited works of the manuscript that the reference search of the authors ended in mid June 2020. No later paper is cited except a WHO guideline. This is unacceptable in this exploding area of research.

Both that introduction and the discussion must include recent comparative meta-analyses on the subject. In this respect, the Discussion should also emphasize the novelty of the present work compared to those.

Bwire GM, Majigo MV, Njiro BJ, Mawazo A. Detection profile of SARS-CoV-2 using RT-PCR in different types of clinical specimens: A systematic review and meta-analysis. J Med Virol. 2021 Feb;93(2):719-725. doi: 10.1002/jmv.26349. Epub 2020 Aug 2. PMID: 32706393; PMCID: PMC7404904.

Mohammadi A, Esmaeilzadeh E, Li Y, Bosch RJ, Li JZ. SARS-CoV-2 detection in different respiratory sites: A systematic review and meta-analysis. EBioMedicine. 2020 Sep;59:102903. doi: 10.1016/j.ebiom.2020.102903. Epub 2020 Jul 24. PMID: 32718896; PMCID: PMC7380223.

Our reply: It is kindly submitted that the authors had already mentioned and discussed the findings of all the research article included in the meta-analysis of Bwire et al like findings of Wang et al, Xu et al, Lo et al, Chan et al, Chen et al, Liu et al, Wang W et al. Further agreeing to your kind suggestion, we have included the specific finding of both these meta-analysis in our discussion. Novelty of the present work also has also been mentioned in revised manuscript mentioned as under.

Among all the published reports, novelty of the present study lies in its assessment of various clinical specimen in both diagnosis and follow-up of COVID-19 patients. To the best of authors’ knowledge, none of the earlier studies evaluated combined swabs as the potential clinical specimen in both diagnosis and monitoring of treatment follow-up cases. Present study found combined swab to provide appropriate clinical picture of clearing of the viruses from the patient undergoing treatment as it was the last specimen among all tested specimen to turned negative. This finding has clinical implication as early negative result of other specimen in follow-up investigation can give pseudoimpression of virus clearance leading to potential risk of transmission of the COVID-19 infection in case if such patients are discharged.

2. Key original papers having similar aims as the present work should also be quoted and compared to the present data in the Discussion:

Liu M, Li Q, Zhou J, Ai W, Zheng X, Zeng J, Liu Y, Xiang X, Guo R, Li X, Wu X, Xu H, Jiang L, Zhang H, Chen J, Tian L, Luo J, Luo C. Value of swab types and collection time on SARS-COV-2 detection using RT-PCR assay. J Virol Methods. 2020 Dec;286:113974. doi: 10.1016/j.jviromet.2020.113974. Epub 2020 Sep 16. PMID: 32949663; PMCID: PMC7493793.

Tong Y, Bao A, Chen H, Huang J, Lv Z, Feng L, Cheng Y, Wang Y, Bai L, Rao W, Zheng H, Wu Z, Qiao B, Zhao Z, Wang H, Li Y. Necessity for detection of SARS-CoV-2 RNA in multiple types of specimens for the discharge of the patients with COVID-19. J Transl Med. 2020 Nov 2;18(1):411. doi: 10.1186/s12967-020-02580-w. PMID: 33138834; PMCID: PMC7605325.

Zhang H, Chen M, Zhang Y, Wen J, Wang Y, Wang L, Guo J, Liu C, Li D, Wang Y, Bai J, Gao G, Wang S, Yang D, Yu F, Yan L, Wan G, Zhang F. The Yield and Consistency of the Detection of SARS-CoV-2 in Multiple Respiratory Specimens. Open Forum Infect Dis. 2020 Aug 26;7(10):ofaa379. doi: 10.1093/ofid/ofaa379. PMID: 33072810; PMCID: PMC7499703.

Rao M, Rashid FA, Sabri FSAH, Jamil NN, Seradja V, Abdullah NA, Ahmad H, Aren SL, Ali SAS, Ghazali M, Manaf AA, Talib H, Hashim R, Zain R, Thayan R, Amran F, Aris T, Ahmad N. COVID-19 screening test by using random oropharyngeal saliva. J Med Virol. 2021 Jan 4. doi: 10.1002/jmv.26773. Epub ahead of print. PMID: 33393672.

**Our reply:** Sincerely acknowledging the kind comment of reviewer, we have incorporated all suggested studies and critically analyzed our result with finding of these studies. In doing so, we have re-framed the introduction and discussion section of the manuscript.

3. The authors suggested that their lower Ct in combined and NPS swab indicated the primary nCoV colonization site is the posterior nasopharynx. They did not provide any direct evidence for this. Therefor this should be removed from the major findings and conclusion. However, the indirect evidence that they provided should be discussed and compared to data of others received by other methodologies.

**Our reply:** As per the suggestion, we have removed our statement of primary nCoV colonization site is the posterior nasopharynx and modified it as indirect evidence in the discussion.

4. The data and the details of Figure 4 are simply invisible. Downloaded high resolution does not help on this a lot. For visibility and clarity, this figure should be completely redrawn.

**Our reply**: We have redrawn the figure 4. Hopefully it will be accessible in high resolution at your end.

5. The limitations of the study should be discussed in the Discussion in more details. For example, saliva is one of the most promising diagnostic sample. This should be discussed. At least the following meta-analysis should be cited and briefly credited in the discussion:

Czumbel LM, Kiss S, Farkas N, Mandel I, Hegyi A, Nagy Á, Lohinai Z, Szakács Z, Hegyi P, Steward MC, Varga G. Saliva as a Candidate for COVID-19 Diagnostic Testing: A Meta-Analysis. Front Med (Lausanne). 2020 Aug 4;7:465. doi: 10.3389/fmed.2020.00465. PMID: 32903849; PMCID: PMC7438940.

**Our reply:** Yes, the limitation of the study has been discussed in more detail in the discussion of revised manuscript.

6. The English language of the paper needs extensive revision by a professional language editor. Particularly, many sentences are very long, complicated, therefore, hard to understand.

Our reply: the English language is revised extensively in the revised manuscript.

6. PLOS authors have the option to publish the peer review history of their article ([what does this mean?](https://journals.plos.org/plosone/s/editorial-and-peer-review-process#loc-peer-review-history)). If published, this will include your full peer review and any attached files.
