# Supplementary material for: Comparative analysis of various clinical specimens in detection of SARS-CoV-2 using rRT-PCR in new and follow up cases of COVID-19 infection: Quest for the best choice
Source: PLoS One. 2021 Apr 5;16(4):e0249408. doi: 10.1371/journal.pone.0249408 (PMC8021168; doi:10.1371/journal.pone.0249408)
Supplement: Attachment — Submitted filename: 2nd Response to reviewer.docx [file pone.0249408.s003.docx]

**Journal grammatical comments and our reply**

**General comment:** While the manuscript is scientifically sound, there are format, typos and awkward sentences through out the manuscript that needs to be corrected (I have attached a file containing examples of my suggestion). We suggest you thoroughly copyedit your manuscript for language usage, spelling, and grammar.

**Reply:** As per the suggestion, we have point wise addressed all the suggestion shown in the attachment. The point wise reply is mentioned below. Moreover, we have also adequately addressed the issue of grammatical addressing of the manuscript. All reframed sentences in the manuscript has been highlighted in red in the revised manuscript.

**Comments 1:** Abstract, Line No. 9 is awkward sentence.

**Reply:** We have reframed the said sentence as “A total of 924 initial specimens from 130 COVID-19 symptomatic cases before initiation of treatment and 665 follow up specimens from 15 randomly selected cases comprising of equal number of nasopharyngeal swab (NPS), oropharyngeal swab (OPS), combined NPS and OPS (Combined swab), sputum, plasma, serum and urine were evaluated by rRT-PCR. Combined swabs showed a positivity rate of 100 % followed by NPS (91.5%), OPS (72.3%), sputum (63%), while nCoV was found undetected in urine, plasma and serum specimens” in the re-revised manuscript.

**Comment 2:** The lowest cycle threshold (Ct) values of 10.56, 10.1415 and 12.26 and lowest average Ct values of targeted genes *E*  were found in combined swab

17 among all specimen types to indicate higher viral load in it.

**Reply:** As per the suggestion, the necessary change has been incorporated in the re-revised manuscript.

**Comment 3:** Serological test based on SARS-CoV-2 antibody detection, have55 been reported with varying sensitivity (34 to 80%), cross reactivity with other SARS-CoV,

56 varying rates of seroconversion between 7 and 11 days after onset of symptoms and varying

57 immunological response by [7,8].

**Reply:** As per the suggestion, the necessary change has been incorporated in the re-revised manuscript.

**Comment 4:** These include upper respiratory tract specimen ( saliva, OPS, NPS, nasal swab),

73 lower respiratory specimen {sputum, bronchoalveolar lavage fluid (BLF) endotracheal

74 aspirate(ET), fibrobronchoscope brush biopsy(FBB)}, blood and its products (serum,

75 plasma), urine, feces and rectal swab.

**Reply:** No its not a BAL. BAL specimen is mentioned in next line as bronchoalveolar lavage fluid (BLF). For better clarity of the text, we have changed the abbreviation of BLF to BAL in the re-revised manuscript.

The reframed sentence will be read as “These include upper respiratory tract specimen (saliva, OPS, NPS, nasal swab), lower respiratory specimen {sputum, bronchoalveolar lavage (BAL), endotracheal aspirate (ET), fibrobronchoscope brush biopsy (FBB)}, blood and its products (serum, plasma), urine, feces and rectal swab.”

**Comment 5:** Page no. 5, line no. 78, define BAL.

**Reply:** As suggested, we have defined BAL earlier in line number 71 of page number 4 in re-revised manuscript.

**Comment 6:** Page number 14, line number 144, “ an equal number of what? male or females?

**Reply:** Yes, the 14 tracheal aspirate were obtained from 14 (equal number of) intubated patients. This needs to specify especially considering the fact that many a time, multiple tracheal aspirate are received from same group of patients.

**Comment 7:** Page number 25: Awkward sentence throughout the manuscript.

**Reply:** We have re-wrote majority of the sentences in the revised manuscript and all changes were highlighted in red.

**Specific comment:** While revising your submission, please upload your figure files to the Preflight Analysis and Conversion Engine (PACE) digital diagnostic tool, <https://pacev2.apexcovantage.com/>. PACE helps ensure that figures meet PLOS requirements. To use PACE, you must first register as a user. Registration is free. Then, login and navigate to the UPLOAD tab, where you will find detailed instructions on how to use the tool. If you encounter any issues or have any questions when using PACE, please email PLOS at [figures@plos.org](mailto:figures@plos.org). Please note that Supporting Information files do not need this step.

Reply: As suggested, we have uploaded all four figures used in the manuscript.
