# Supplementary material for: Comparative analysis of various clinical specimens in detection of SARS-CoV-2 using rRT-PCR in new and follow up cases of COVID-19 infection: Quest for the best choice
Source: PLoS One. 2021 Apr 5;16(4):e0249408. doi: 10.1371/journal.pone.0249408 (PMC8021168; doi:10.1371/journal.pone.0249408)
Supplement: Attachment — Submitted filename: Response to Reviewer.docx [file pone.0249408.s005.docx]

**Journal academic editor comments and our reply**

**Dear Editor, since some of the suggested changes in the PDF attachment has only shown symbolically, we have here wrote these suggestion in terms of comments. The appropriate reply to these changes has also been mentioned below.**

**Comment 1:** Abstract, line no. 13: Replace ‘of’ by are and add ‘respectively’ after 12.26.

**Our reply:** The necessary changes has been incorporated in the revised manuscript.

**Comment 2:** Abstract, line no. 18: Create space after ‘average’.

**Our reply:** The necessary change has been incorporated in the revised manuscript.

**Comment 3:** Introduction, line no. 27, nCoV has already defined in abstract and not need to repeat.

**Our reply:** As suggested, necessary changes has been done in the revised manuscript.

**Comment 4:** Page no. 9, line no. 176, create space after ‘version 18’.

**Our reply:** As suggested, necessary changes has been done in the revised manuscript.

**Comment 5:** Page no. 10, Table 2, incorporate changes as indicated.

**Our reply:** As suggested, necessary changes has been done in the revised manuscript.

**Comment 6:** Page no. 11, Line no. 210-212, incorporate the changes as indicated.

**Our reply:** As suggested, necessary changes has been done in the revised manuscript.

**Comment 7:** Page no. 11, Line no. 231, Delete ‘ longer’.

**Our reply:** As suggested, we have deleted the word ‘ longer’ in the revised manuscript.

**Comment 8:** Page no. 11, Line no. 231, change ORF1b to italic.

**Our reply:** As suggested, necessary changes has been done in the revised manuscript.

**Comment 9:** Page 12, line no. 235, incorporate the changes as indicated.

**Our reply:** The necessary changes has been done in the revised manuscript.

**Comment 10:** Page no. 24, Reference 29, “Need more detail information of the reference”.

**Our reply:** Yes, the detail information of the reference number 29 has been incorporated in the revised manuscript.
